# Well-Ordered Trimeric HIV-1 Subtype B and C Soluble Spike Mimetics Generated by Negative Selection Display Native-like Properties

**DOI:** 10.1371/journal.ppat.1004570

**Published:** 2015-01-08

**Authors:** Javier Guenaga, Natalia de Val, Karen Tran, Yu Feng, Karen Satchwell, Andrew B. Ward, Richard T. Wyatt

**Affiliations:** 1 IAVI Neutralizing Antibody Center at The Scripps Research Institute, La Jolla, California, United States of America; 2 Department of Integrative Structural and Computational Biology, The Scripps Research Institute, La Jolla, California, United States of America; 3 Center for HIV/AIDS Vaccine Immunology and Immunogen Discovery, The Scripps Research Institute, La Jolla, California, United States of America; 4 Department of Immunology and Microbial Science, The Scripps Research Institute, La Jolla, California, United States of America; Institut Pasteur, France

## Abstract

The structure of BG505 gp140 SOSIP, a soluble mimic of the native HIV-1 envelope glycoprotein (Env), marks the beginning of new era in Env structure-based immunogen design. Displaying a well-ordered quaternary structure, these subtype A-derived trimers display an excellent antigenic profile, discriminating recognition by broadly neutralizing antibodies (bNAbs) from non-broadly neutralizing antibodies (non-bNAbs), and provide a solid Env-based immunogenic platform starting point. Even with this important advance, obtaining homogeneous well-ordered soluble SOSIP trimers derived from other subtypes remains challenging. Here, we report the “rescue” of homogeneous well-ordered subtype B and C SOSIP trimers from a heterogeneous Env mixture using CD4 binding site-directed (CD4bs) non-bNAbs in a negative-selection purification process. These non-bNAbs recognize the primary receptor CD4bs only on disordered trimers but not on the native Env spike or well-ordered soluble trimers due to steric hindrance. Following negative selection to remove disordered oligomers, we demonstrated recovery of well-ordered, homogeneous trimers by electron microscopy (EM). We obtained 3D EM reconstructions of unliganded trimers, as well as in complex with sCD4, a panel of CD4bs-directed bNAbs, and the cleavage-dependent, trimer-specific bNAb, PGT151. Using bio-layer light interferometry (BLI) we demonstrated that the well-ordered trimers were efficiently recognized by bNAbs and poorly recognized by non-bNAbs, representing soluble mimics of the native viral spike. Biophysical characterization was consistent with the thermostability of a homogeneous species that could be further stabilized by specific bNAbs. This study revealed that Env trimers generate different frequencies of well-ordered versus disordered aberrant trimers even when they are genetically identical. By negatively selecting the native-like well-ordered trimers, we establish a new means to obtain soluble Env mimetics derived from subtypes B and C for expanded use as candidate vaccine immunogens.

## Author Summary

The HIV envelope glycoprotein (Env) is the sole virally encoded gene product on the surface of the virus and, as such, is the only target of neutralizing antibodies. A broadly efficacious HIV vaccine will likely need to generate a robust neutralizing antibody response directed at conserved elements of the variable Env. For a successful antibody-based vaccine, a soluble mimic of the HIV spike will likely be required to generate high-titer anti-Env antibodies capable of neutralizing a wide array of HIV isolates. Due to the global sequence diversity of Env, generating a diverse array of these soluble spikes will benefit immunization strategies designed to cope with such viral diversity. Here, we report a novel purification strategy followed by a comprehensive characterization of two soluble HIV spikes from infection-prominent subtypes, B and C. We demonstrate that these homogeneous soluble trimers are faithful mimics of the HIV spike by neutralizing antibody binding, electron microscopy and other biophysical assessments. Possessing soluble and stable mimics of the HIV spike derived from diverse strains improves both our knowledge of HIV spike architecture as shown here and extends subtype coverage of potential vaccine candidates.

## Introduction

The HIV-1 envelope glycoprotein (Env) is a trimer of heterodimers composed of two non-covalently associated subunits: the receptor-binding gp120 and the fusion machinery-containing gp41. Each subunit is derived from a gp160 precursor glycoprotein following cleavage by cellular furins [1]. HIV-1 gp120 binds the CD4 molecule on the surface of human target T cells to initiate the viral entry process, and following co-receptor engagement, fusion is mediated by gp41 [2]–[4]. The surface-exposed HIV-1 Env trimer is the sole target for antibodies capable of neutralizing the virus [5]. Recently, a myriad of Env-directed broadly neutralizing antibodies (bNAbs) were isolated from numerous HIV-1-infected individuals, demonstrating that the human B cell response can effectively inhibit this variable pathogen [6]–[11]. Infection of macaques by a chimeric model virus, SHIV, can be prevented by prior passive immunization of all bNAbs so far tested, confirming the capacity of neutralizing antibodies to prevent HIV infection [12]–[15].

Along with virus-specific T cells, an efficacious HIV-1 vaccine therefore would likely need to generate bNAbs targeting Env. Although the premise is simple, in actuality, it is a tremendous challenge without precedent in the history of vaccinology. The difficulty to vaccinate against HIV arises from the extensive variability of Env present on the large number of HIV-1 isolates simultaneously circulating in the human population as well as other mechanisms of immune evasion selected for by strong pressure from the human immune system.

Generally, vaccine-generated antibodies using either or both gp120 or gp41 sequences do not recognize native Env on the surface of cells or virus, do not neutralize primary isolates *in vitro*, and do not prevent infection in laboratory animals [16]–[18]. Non-neutralizing antibodies directed to the major variable region two (V2) of gp120 are associated with modest efficacy in a single human clinical trial [19], [20], while, in general, Env-elicited antibodies fail to demonstrate protection in previous human clinical trials [21]–[23].

Many Env-based trimeric candidate immunogens are engineered to eliminate cleavage between gp120 and gp41 (so called uncleaved gp140 trimers), usually generating imperfect mimetics of the functional spike based on antigenic profiling or EM analysis [18], [24]. As a group, the defined, or presumed to be, disordered trimers (in adjuvant) generate high self-binding antibody titers. However, these vaccine-elicited antibodies do not efficiently neutralize most HIV-1 primary isolates, that is, strains representative of those circulating in the human population [17], [25]–[27]. Antibodies elicited by these immunogens target epitopes exposed only on the free gp120 and trimeric post-fusion forms of gp41 or disordered gp140s and thus are ineffective at accessing their epitopes buried within the ordered, quaternary structure achieved in the native Env spike. We recently described the limitations of two CD4 binding site (CD4bs)-directed non-bNAbs, (GE148 and GE136) generated following immunization of uncleaved gp140 trimers (YU2 gp140-foldon) in non-human primates (NHP). Non-bNAbs, represented by GE136 and GE148, can only neutralize the sensitive so-called “tier 1 viruses” that are not representative of the more neutralization resistant tier 2 primary isolates circulating in the human population. Using crystallography, EM reconstructions, paratope scanning and molecular modeling we determined that these vaccine-elicited antibodies fail to reach the CD4bs due to steric barriers imposed by quaternary packing of the native Env on neutralization resistant primary isolates, a property that we use to our advantage in the negative-selection strategy presented here [18].

The cumulative historical data have led to the hypothesis that a more faithful mimic of the HIV-1 spike that better recapitulates the native, pre-fusion form of Env, selectively displaying neutralizing determinants while occluding non-neutralizing determinants, may better elicit antibodies capable of accessing the native spike. A soluble Env mimetic, containing a disulfide linkage between gp120 and gp41 (SOS), first described in the 2000 s, and further developed over the next decade, displays many of these properties, culminating in the determination of the high resolution structures of the well-ordered BG505 SOSIP trimers by crystallography and EM [28]–[31]. A sub-nanometer EM reconstruction of KNH1144 SOSIP is also available but does not provide atomic level details [32]. The BG505 SOSIP and KNH1144 SOSIP trimers are derived from the Env sequences of the subtype A BG505 and KNH1144 strains. These soluble trimers possess an engineered disulfide linkage between the gp120 and gp41 (at residues 501C and 605C, respectively) and an additional mutation in the heptad repeat 1 (HR1) of gp41 (I559P) that facilitates trimerization [33], [34]. A truncation of the membrane proximal external region (MPER) at residue 664 that enhances expression while decreasing aggregation is incorporated into the so-called BG505 SOSIP.664 trimers [30], [35]. Although SOSIP molecules based on other HIV-1 primary strains were attempted over the past decade, the BG505- and KNH1144-derived SOSIP trimers are the two limited examples of SOSIPs that yield homogeneous trimers suitable for high resolution biophysical and structural analysis. The structural explanation for the difficulty to readily transfer the SOSIP design to other HIV-1 strain-derived sequences is not yet fully understood and would be valuable information to broaden the trimer design horizon.

Here, we describe two SOSIP trimer molecules derived from the B subtype strain, JRFL, and the subtype C strain, 16055. We selected these two Envs for the initial results reported in this study as follows. The JRFL SOSIP trimer, truncated at residue 663 (JRFL SOSIP) derives from the JRFL HIV-1 strain isolated from the frontal lobe (FL) of an HIV-1-infected individual. This Env is often used because it displays the unusual property that its gp160 Env precursor is efficiently cleaved into the gp120 and gp41 subunits when expressed on the cell surface of 293F HEK cells [36]. The 16055 SOSIP trimer, also truncated at residue 663, derives from a HIV-1 Indian strain and displays the unusual property that its monomeric gp120 is weakly recognized by the quaternary epitope-preferring bNAbs, PG9 and PG16, which is relatively infrequent amongst most HIV-1 Env sequences [37], and is also observed for BG505 gp120 [38], [39].

In the current study, we demonstrate that the JRFL and 16055 SOSIP trimers were purified to homogeneity by a novel means of isolation that utilizes non-bNAbs targeting the CD4bs in a negative-selection process that effectively separates well-ordered trimers from a mixture also containing disordered aberrant trimers and other oligomeric states of Env. By bio-layer light interferometry (BLI) binding analysis, we demonstrated that the purified JRFL and 16055 SOSIP trimers were efficiently recognized by bNAbs but were poorly recognized by the non-bNAbs. By negative stain EM, we confirmed that negative selection results in homogeneous, well-ordered JRFL and 16055 SOSIP trimers displaying a 3-lobed architecture resembling the native HIV spike and the previously described subtype A SOSIP trimers. We obtained 3D EM reconstructions of the unliganded and liganded JRFL and 16055 SOSIP trimers and demonstrated that the negatively selected trimers adopt conformational changes upon sCD4 engagement that emulate those of the native HIV spike [40]. Differential scanning calorimetry (DSC) and differential scanning fluorimetry (DSF) revealed that the negatively selected JRFL and 16055 SOSIP trimers were stable at relatively elevated temperatures, exhibiting melting temperatures (T_m_) exceeding 58 and 63°C, respectively. We conclude that the negative-selection process resulted in highly homogenous well-ordered JRFL and 16055 trimers, expanding the SOSIP family of Env mimetics to HIV-1 subtypes B and C. This advance provides opportunities for HIV Env structural comparisons at high resolution as well as a wider array of ordered trimers for sequential or simultaneous inoculation regimens to evaluate enhanced immunogenicity toward more broadly effective antibody responses.

## Results

### Purification of the SOSIP trimers by negative selection results in well-ordered trimers

**Figure 1:**
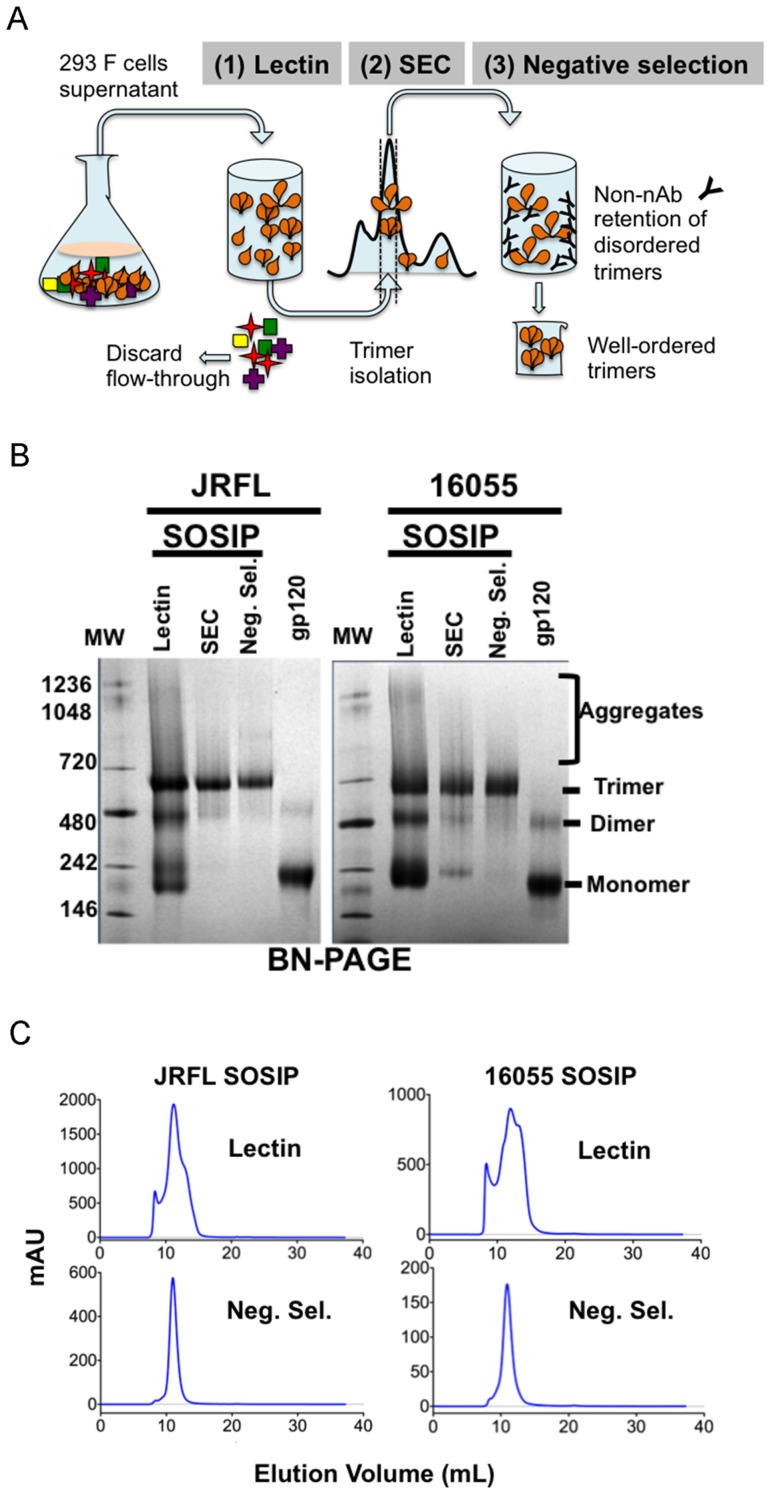
The SOSIP purification strategy. (A) 293F cells were co-transfected with plasmid DNA encoding both SOSIPs and furin sequences and cultured for 5–6 days before collection of supernatants. Filtered supernatants were flowed over a lectin affinity chromatography column and the eluate containing the over-expressed soluble SOSIP molecules were resolved by SEC to isolate the trimer-containing fractions. Lastly, these fractions were passed over a protein A column immobilized with F105 for JRFL, or GE136 for 16055, to negatively select the disordered oligomers from the mixture. Disordered oligomers are retained on the column from the well-ordered trimers that flow through the column unimpeded. (B) Blue-native gel analysis of JRFL and 16055 gp120 monomers and SOSIP oligomers. Molecular weight markers (MW) are in the first lane, the lectin-purified SOSIP oligomers are in the second lane, revealing three prominent bands corresponding to trimeric, dimeric and monomeric SOSIP forms. The third lane labeled “SEC” contains one predominant band corresponding to trimeric SOSIP forms, including well-ordered and disordered trimers. Faint bands corresponding to dimer and monomer contaminants were detected. The fourth lane containing negative-selected samples (“Neg. Sel.”) displayed predominantly one band corresponding to the expected size of the SOSIP trimer. The last lane contains the monomeric gp120 control and faint band corresponding to gp120 dimers. (C) Top panels depict SEC profiles of the lectin-purified JRFL and 16055 SOSIP samples. JRFL displayed a dominant peak, corresponding to the trimeric form and a smaller shoulder to the right of the peak corresponding to dimeric and monomeric forms of SOSIP. The 16055 SOSIP trimer, dimer and monomer peaks are overlapping. Bottom panels display negatively selected SOSIP trimer samples as a homogeneous highly symmetric peak.

As an overall approach, the JRFL SOSIP and 16055 SOSIP trimer glycoproteins, designed on the established SOSIP template as described in Methods and S1A Fig., were purified in three steps consisting of lectin-affinity chromatography, followed by size exclusion chromatography (SEC), followed by a final negative-selection procedure (Schematic, Fig. 1A). Blue native polyacrylamide gel electrophoresis (BN-PAGE) analysis of the lectin-purified proteins revealed bands corresponding to the expected size of SOSIP trimers, along with undesired monomers, dimers and higher-order oligomeric forms. The distribution of oligomers was slightly different for JRFL SOSIP than for 16055 SOSIP. The JRFL SOSIP glycoproteins presented as a predominant band corresponding to the trimeric species with lower intensity bands corresponding to dimeric and momeric forms. In contrast, 16055 SOSIP displayed bands of similar magnitude for all oligomeric forms detected (Fig. 1B). The lectin-purified glycoproteins of both SOSIP types were subjected to SEC and the corresponding chromatograms corroborated the distribution of oligomeric forms observed by BN-PAGE. Specifically, the JRFL SOSIP protein peak corresponding to the trimeric form of SOSIP eluted at approximately 11 ml, with a shoulder at 12 ml, corresponding to dimers, with a smaller peak at 13 ml corresponding to monomers (Fig. 1C). The 16055 SOSIP SEC profile showed three overlapping peaks of similar magnitude, suggesting a less efficient tendency of this Env to form SOSIP trimers compared to JRFL (Fig. 1C). Elution fractions containing the expected trimers (elution volume 10–12 ml) were collected and contained primarily trimers along with associated dimers and monomers that could not be resolved by SEC.

**Figure 2:**
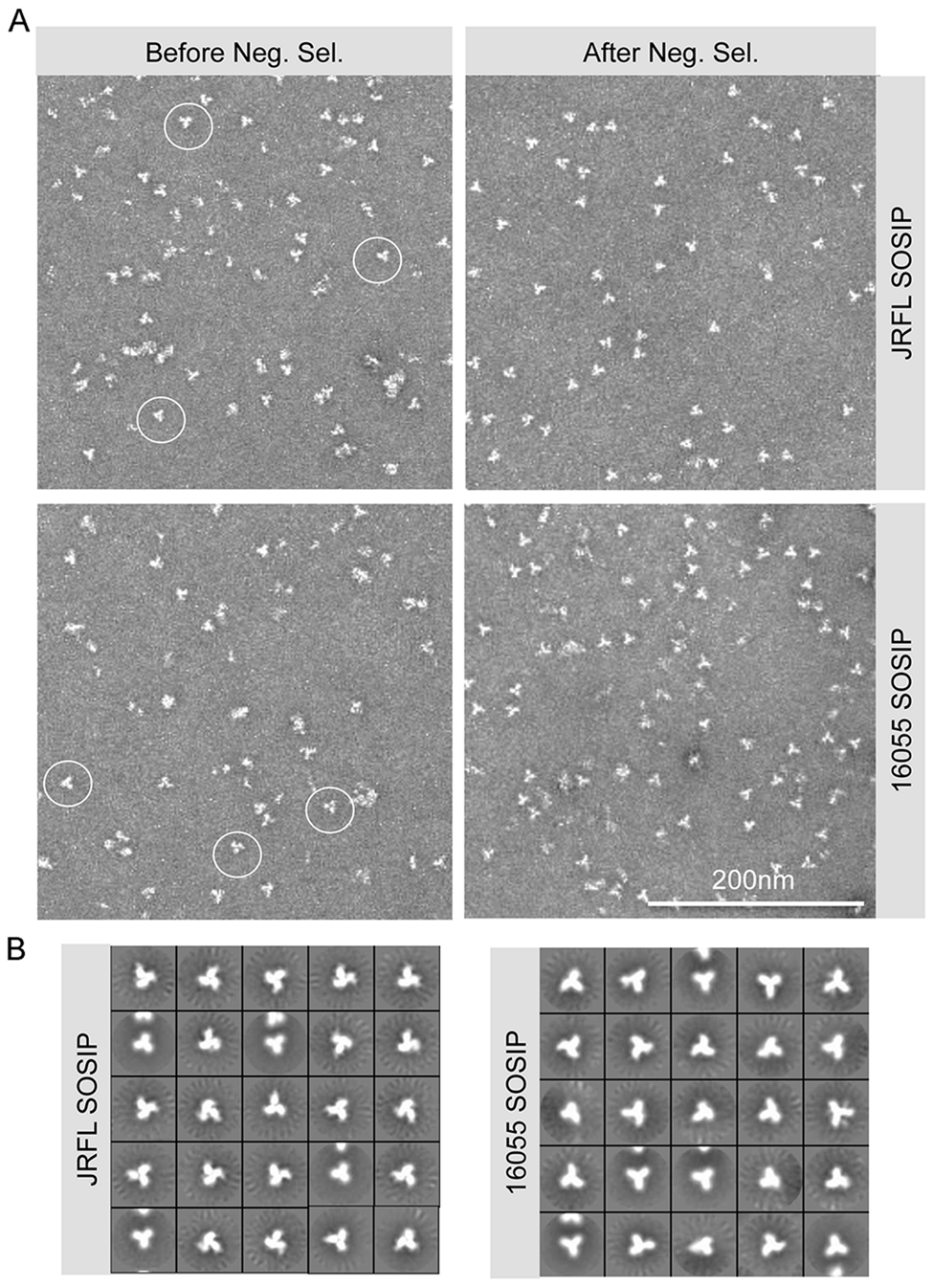
SOSIP EM micrographs and 2D class averages. (A) Within the SEC isolated trimer fraction, disordered oligomers along with well-ordered trimeric proteins (circled) are detected (left). Approximately 60% of the trimers are well-ordered before negative selection. The negatively selected samples display a majority of well-ordered trimeric proteins (right). (B) EM 2D class averages of SOSIP trimers after negative selection.

With the goal of resolving the mixture of SOSIP oligomeric states, we reasoned that the CD4bs-directed non-bNAbs GE136 or GE148 might be able to absorb out disordered trimers, dimers and monomers. We also included the similar, but infection-elicited CD4bs-directed non-bNAb, F105, in our analysis. Each of these non-bNAbs inefficiently target the CD4bs on the trimeric HIV spike with a vertical angle of approach that clashes with the variable region cap on the well-ordered native spikes [18] and therefore do not neutralize either JRFL or 16055 virus. We sought to use these CD4bs-directed non-bNAbs to remove the disordered, and presumably more conformationally open, trimers, dimers and monomers from the oligomeric mixture in a negative-selection purification step. To begin, we first assessed which non-bNAb most efficiently immuno-precipitated the unresolved Env forms from the mixture, selecting F105 for JRFL and GE136 for 16055, which displayed favorable binding kinetic parameters for the respective gp120s (S1B Fig.). F105 did not efficiently immuno-precipitate 16055 Env forms and also displayed a faster dissociation rate for 16055 gp120 (S1B Fig.). Subsequently, lectin- and SEC-purified SOSIP samples were flowed over protein A affinity columns with F105 or GE136 previously immobilized on this matrix. Analyzing the flow-through from the affinity-column, we observed that the SOSIP trimers migrated as a highly homogenous single peak by SEC, suggesting that the negative-selection approach removed aggregates, dimers, and monomers (Fig. 1C). Negative selection retained disordered trimers, dimers and monomers on the solid phase, presumably by allowing the non-bNAbs F105 or GE136 access to the CD4bs on these aberrant forms of Env. This retention is readily apparent for 16055, where the disappearance of the dimer and monomer bands on the BN-PAGE gel can be observed following negative selection compared to before (Fig. 1B). BN-PAGE analysis of the lectin- and SEC-purified, negatively selected JRFL and 16055 SOSIP trimers revealed a single band corresponding to the expected size of a trimeric SOSIP protein (Fig. 1B). To confirm the effectiveness of the separation process, we examined the SOSIP samples before and after the negative-selection affinity chromatography process by negative stain EM. Visual inspection of the EM micrographs showed a pronounced reduction of aberrant or disordered SOSIP oligomers following negative selection (Fig. 2A). Negative selection yielded highly homogeneous well-ordered native-like trimers in the respective eluates based on EM 2D classification (Fig. 2B). Yields after negative selection were typically 1.5 mg per liter for JRFL SOSIP and 0.5 mg per liter for 16055 SOSIP. Negative selection removed approximately 50-60% of disordered forms of Env from the SEC trimer-isolated fraction. As expected, reducing SDS-PAGE revealed that both negatively selected JRFL and 16055 SOSIP trimers appeared predominantly as two bands on the gel, corresponding to Env glycoprotein subunits gp120 and gp41, indicating effective furin cleavage of the SOSIP trimers (S1C Fig.).

### Antigenic analysis of JRFL and 16055 SOSIP trimers by bio-layer interferometry (BLI) confirms recognition by bNAbs

**Figure 3:**
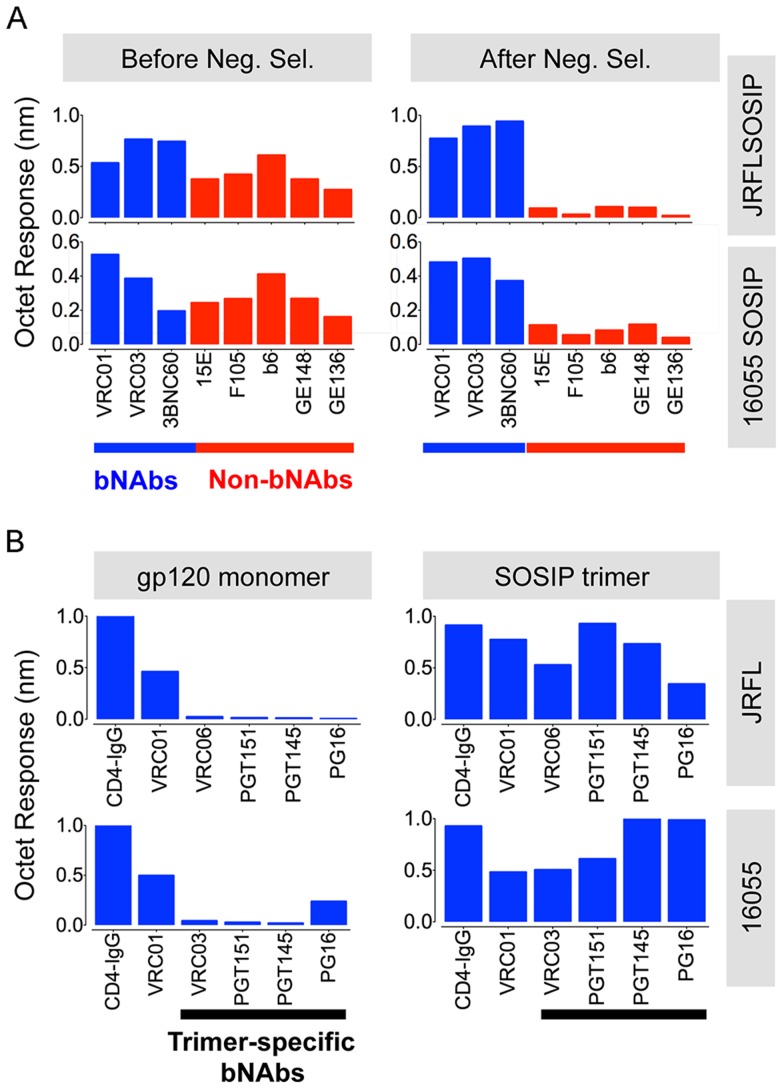
Negative selected SOSIP trimers display a favorable antigenic profile. (A) Bars represent BLI (Octet) maximal response values derived from binding curves (S2 Fig. and S3 Fig.). These Octet curves were obtained with the SOSIP trimers (200 nM) as the analyte in solution and a panel of well-characterized anti-HIV-1 Env monoclonal antibodies immobilized on anti-human IgG Fc sensor tips. The blue bars represent BLI response values obtained by using bNAbs whereas the red bars denote BLI response values generated by non-bNAbs, before (left panels) and after (right panels) the process of negative selection. (B) Binding of selected panel of bNAbs to monomeric gp120 (600 nM) in comparison with negatively selected SOSIP trimer (200 nM). These response values were derived from binding curves shown in S2 Fig. and S3 Fig.

We next investigated the effect of negative selection on trimer antigenicity using a set of CD4bs-directed bNAbs and non-bNAbs. We employed BLI (Octet) to assess mAb binding to JRFL and 16055 SOSIP trimers in solution, before and after negative selection. To begin the analysis, we plotted the BLI maximal response values from binding curves as a bar graph, permitting a semi-quantitative relative assessment of binding for each antibody. We observed that negative selection eliminated nearly all recognition of JRFL and 16055 SOSIP trimers by the CD4bs-directed non-bNAbs (“F105-like”), compared to before negative selection (Fig. 3A, S2A Fig. and S3A Fig.).

**Figure 4:**
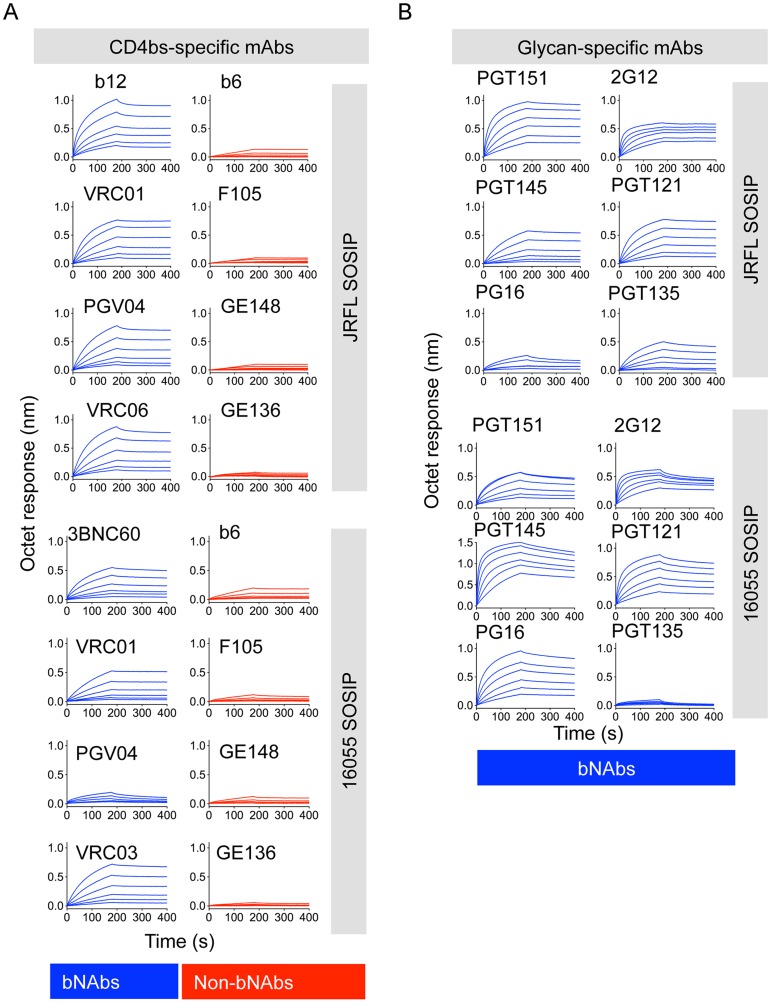
Bio-layer interferometry (BLI) analysis of CD4bs- and glycan- directed mAbs. (A) BLI curves generated with a panel of CD4bs-directed antibodies immobilized on anti-human IgG Fc sensors and a serial dilution (200 nM–6.25 nM) of negatively selected SOSIP trimers in solution as the analyte. (B) Similar BLI curves obtained with glycan-directed bNAbs.

Assessing trimer recognition by quaternary epitope preferring bNAbs such as PG16, PGT151, PGT145, VRC06 and VRC03 was especially useful for this antigenic analysis. Their trimer recognition, or lack thereof, provided a means to discern between a native-like, well-ordered trimer and an “open” disordered conformation of the trimer. The glycan-specific bNAbs PG16, PGT145 and PGT151 are known for their quaternary epitope specificity and, as such, they do not efficiently recognize monomeric gp120 (Fig. 3B, S2A Fig. and S3A Fig.) [8], [41]–[43]. The CD4bs-directed VRC03 and VRC06, unlike VRC01, possess a framework region insertion that extends their paratope beyond the CD4bs into the V3 loop of the adjacent protomer (within a trimer) conferring upon them a trimer-preferring character [28], [44]. VRC06 and VRC03 did not recognize gp120 while the non-trimer preferring control mAbs, VRC01 and CD4-IgG, did recognize the soluble monomer (Fig. 3B, S2A Fig. and S3A Fig.). In fact, all of these trimer-preferring antibodies showed increased recognition of SOSIP trimers, as compared with their recognition of monomeric gp120 (Fig. 3B, S2A Fig. and S3A Fig.). The preferential recognition by bNAbs following negative selection suggested that this process efficiently eliminated disordered oligomers, consistent with the EM analysis (Fig. 4A).

We also assessed binding of bNAbs 2G12, PGT121, and PGT135 that target an array of glycans clustered around the N332 glycan. While the subtype B JRFL isolate is naturally glycosylated at the N332 site, the subtype C 16055 Env lacks this N-linked glycan. JRFL SOSIP was robustly recognized by all glycan-dependent bNAbs tested, whereas 16055 SOSIP trimers was poorly recognized by the mAb PGT135 (Fig. 4B, S2A Fig. and S3A Fig.). 2G12 and PGT121 binding remained relatively strong, despite faster off-rates compared to JRFL SOSIP, suggesting that the latter two antibodies may use other glycans that compensate for the missing 16055 332 N-linked glycan, as recently suggested for PGT121 (Fig. 4B) [45]. PGT121 is the only antibody that we tested targeting this N332 glycan “site of vulnerability” that neutralizes the HIV-1 subtype C strain, 16055 (S1 Table).

**Figure 5:**
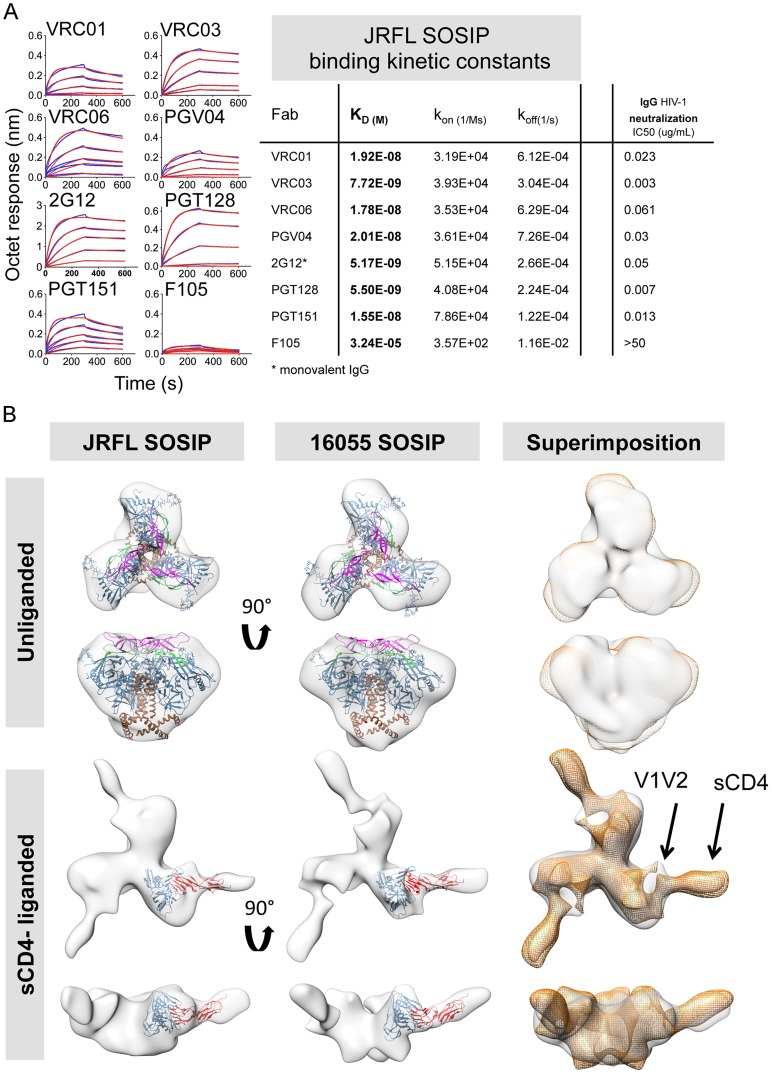
Kinetic measurements and EM 3D reconstructions of unliganded and sCD4-liganded SOSIP trimers. (A) Binding kinetic measurements were determined for a selected panel of antibodies. Corresponding Fabs were used as analytes in solution in a concentration series (400 nM to 25 nM) while the negatively selected JRFL SOSIP trimers were immobilized on anti-His biosensor tips. JRFL HIV-1 neutralization IC50 values of the corresponding IgGs are shown to the left of the JRFL SOSIP binding kinetic constants. (B) Top panels display top and side views of the 3D reconstruction EM densities of unliganded JRFL and 16055 SOSIP trimers in gray with the BG505 SOSIP.664 EM structure (PDB ID 3J5M, gp120 in blue, V1V2 in magenta, V3 in green and gp41 in brown) fitted within. Bottom panel displays top and side views of the four-domain soluble CD4-liganded SOSIP trimers with the sCD4-bound gp120 core crystal structure (PDB ID 1GC1, gp120 in blue and sCD4 in red) fitted in the EM density. To the right, top and side views of the 16055 SOSIP EM density (orange) superimposed over the JRFL SOSIP EM density (in gray). The 2D class averages and Fourier Shell Correlation (FSC) are included in the supporting information.

Additionally, the CD4bs-specific bNAb PGV04, recently used to determine the high-resolution cryo-EM structure of the BG505 SOSIP trimer [28], and b12, both efficiently recognized the JRFL SOSIP trimer, but not the 16055 SOSIP trimer (S2 Fig. and S3 Fig.). Consistent with this differential binding, PGV04 and b12 neutralize the parental JRFL HIV strain while they do not neutralize the 16055 clade C strain in a TZM-bl assay. (S1 Table). Utilizing Fabs derived from a selected panel of antibodies, we determined kinetic constants for their interaction with the JRFL SOSIP trimer His-captured on the biosensor. All neutralizing antibodies tested in this minipanel had affinities ranging from 5 nM to 20 nM, while the non-neutralizing mAb F105 inefficiently recognized the negatively selected trimers with micromolar affinity (Fig. 5A). With a few exceptions, binding of the SOSIP trimer was associated with a neutralizing phenotype of the antibody for the parental HIV strain.

Non-neutralizing mAbs targeting other Env sites, such as 17b, C11, 7b2, did not recognize the negatively selected JRFL and 16055 SOSIP trimers. The V3-directed non-bNAb 19b did recognize the SOSIP trimers even after F105 or GE136 negative selection (S2 and S3 Fig.). By EM, the 19b Fab infrequently bound the negatively selected JRFL or 16055 SOSIP, demonstrating binding by only one Fab to ∼20–25% of trimers, respectively (S4A Fig. and S1 Table). This occupancy was comparable to that displayed by the BG505 SOSIP.664 where 30% of the trimers bound one V3-directed Fab [30]. Recognition by the V3 antibodies as assessed by BLI with the multivalent SOSIP trimers as an analyte, may increase the sensitivity of detection due to avidity effects that are eliminated with the Fab in the EM context.

### Analysis of JRFL AND 16055 SOSIP trimers by EM reveals well-ordered trimers

We obtained 3D reconstructions of the SOSIP trimers in the unliganded state by EM negative stain (Fig. 5B). The overall morphology of the unliganded JRFL and 16055 SOSIP trimers at 21 Å and 18 Å resolution, respectively, is similar to that previously described for BG505 SOSIP.664. All trimers display three-lobed structures and an overall density that is wider at the top and narrower at the bottom corresponding with the association of three gp120 units at the top of the spike and three gp41 units near the bottom (Fig. 5B) [30]. We fitted the cryo-EM derived model of BG505 SOSIP.664 (PDB 3J5M) within the JRFL and 16055 SOSIP EM reconstructions to demonstrate that no gross differences were observed. A superimposition of the unliganded JRFL and 16055 SOSIP densities revealed small differences when comparing their surface contours (Fig. 5B, right panel). These differences may be in part due to the low resolution of the reconstructions. We do however note that there are differences in the glycosylation patterns of the two trimers, as the 16055 HIV-1 Env possesses 28 glycosylation sites while JRFL Env has 25 glycosylation sites, and that difference may partially account for the observed surface contour variation.

We compared the unliganded state to complexes with a soluble version of the HIV-1 primary receptor, soluble four-domain CD4 (sCD4) (Fig. 5B, lower panels). The 3D reconstructions of the JRFL and 16055 SOSIP trimers liganded with sCD4 at 21 Å and 23 Å resolution, respectively, show conformational changes in agreement with cryo-EM images of the sCD4-liganded native BaL Env and the previously published KNH1144 SOSIP:CD4 complexes (S4B Fig.) [40], [46]. During natural infection, such conformational changes presumably follow the engagement of the cellular receptor, CD4, to form or expose the co-receptor binding site. By EM analysis, CD4-induced conformational changes result in the lateral movement of the gp120 subunits and the appearance of a protrusion attributed to the displacement of the V1V2 loops (Fig. 5B and S4B Fig.) [40], [46]. Also, in the current analysis of the JRFL and 16055 soluble spike mimetics, the putative gp41 unit density, located at the bottom of the trimer, opened and flattened when in complex with sCD4 (Fig. 5B). As expected from previous results, CD4 engagement did not abrogate trimerization of the SOSIP trimers despite the large conformational changes observed and despite the truncation of the MPER in these constructs. sCD4 displayed the same angle of approach to the CD4bs in both CD4-liganded JRFL and 16055 SOSIP trimers, consistent with the previous KNH1144 SOSIP-sCD4 and BaL-Env EM analysis (Fig. 5B and S4B Fig.) [40], [46].

**Figure 6:**
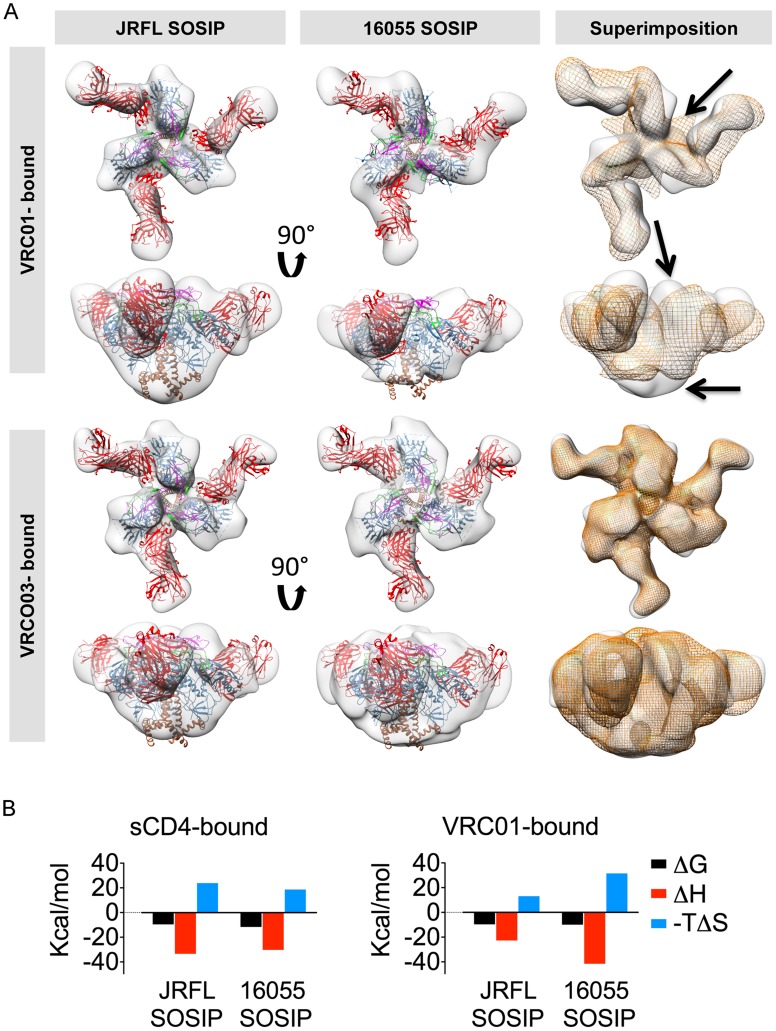
EM 3D reconstructions of VRC01- and VRC03-liganded SOSIP trimers. (A) Top and side views of the 3D reconstruction EM densities of the VRC01-liganded (top) and VRC03-liganded (bottom) JRFL and 16055 SOSIP trimers in gray with the high resolution cryo-EM structure of the PGV04-liganded BG505 SOSIP (PDB 3J5M, gp120 in blue with V1V2 in magenta, V2 in green, gp41 in brown and the PGV04 Fab in red) fitted within. To the right, top and side views of the liganded 16055 SOSIP EM density (orange) superimposed onto the liganded JRFL SOSIP (gray). Arrows indicate differences between the EM densities of the two VRC01-liganded trimers. (B) Comparison of thermodynamic parameters, represented here as bar graphs, resulting from ITC measurements obtained when SOSIP trimers interact with sCD4 and VRC01.

We investigated if the bNAb VRC01, which also targets the CD4bs, resulted in similar quaternary conformational changes in the trimer architecture as those induced by sCD4. Accordingly, we obtained EM 3D reconstructions of VRC01-liganded JRFL and 16055 SOSIP at 20 Å and 22 Å resolution, respectively (Fig. 6A). VRC01 did not induce any apparent conformational changes in the overall architecture of the JRFL SOSIP trimer at the resolution obtained in this study. However, we did detect conformational changes induced by VRC01 interaction with the 16055 SOSIP trimers. These conformational changes were not as pronounced as those induced following engagement with sCD4, however, substantial differences between JRFL and 16055 were observed in the superimposition of the two complexes (Fig. 6A). Specifically, VRC01 adopts an apparent more horizontal angle of approach when bound to 16055 SOSIP trimers as compared to its interaction with the JRFL SOSIP trimers. This angle difference is likely due to a more “open” state of the 16055 SOSIP trimer induced by VRC01 rather than a difference in the interaction of the antibody with its epitope on the corresponding gp120 subunits. The VRC01-bound 16055 SOSIP reconstruction displayed differences in the gp41 subunit that resembled those induced by sCD4 (Fig. 6A and Fig. 5A). To investigate further differential conformational changes induced by VRC01 Fab on the JRFL versus 16055 SOSIP trimers, we performed isothermal titration calorimetry (ITC). We detected much larger favorable enthalpy and unfavorable entropy changes induced by VRC01 Fab in complex with the 16055 SOSIP trimers relative to the JRFL SOSIP trimers, consistent with the EM analysis (Fig. 6B and S5 Fig.). In contrast, ITC parameters assessed with sCD4 were similar for both trimers (Fig. 6B and S5 Fig.).

Next, we investigated if the conformational flexibility exhibited by the 16055 SOSIP trimer was a result of the interaction with VRC01 or an intrinsic property encoded by the 16055 primary sequence and subsequent quaternary assembly. We used VRC03, a VRC01-related bNAb also targeting the CD4bs that, unlike VRC01, showed preferential binding to the trimer relative to the monomer by BLI (Fig. 3B, S2A Fig. and S3A Fig.). We obtained 3D reconstructions of JRFL and 16055 SOSIP trimers in complex with VRC03 at 20 Å and 19 Å resolution, respectively, revealing that VRC03 binding did not result in any apparent conformational changes in either of the SOSIP trimers (Fig. 6A). Unlike the VRC01 densities, the superimposition of the two VRC03-bound trimer densities was highly concordant and the angle of approach of VRC03 was the same for both JRLF and 16055 SOSIP trimers. Taken together, these data suggest that the conformational changes observed in 16055 SOSIP upon VRC01 binding may be induced by the interaction of the mAb with these soluble trimers and not due to increased flexibility of the 16055 SOSIP trimers themselves, consistent with the ITC data (Fig. 6).

**Figure 7:**
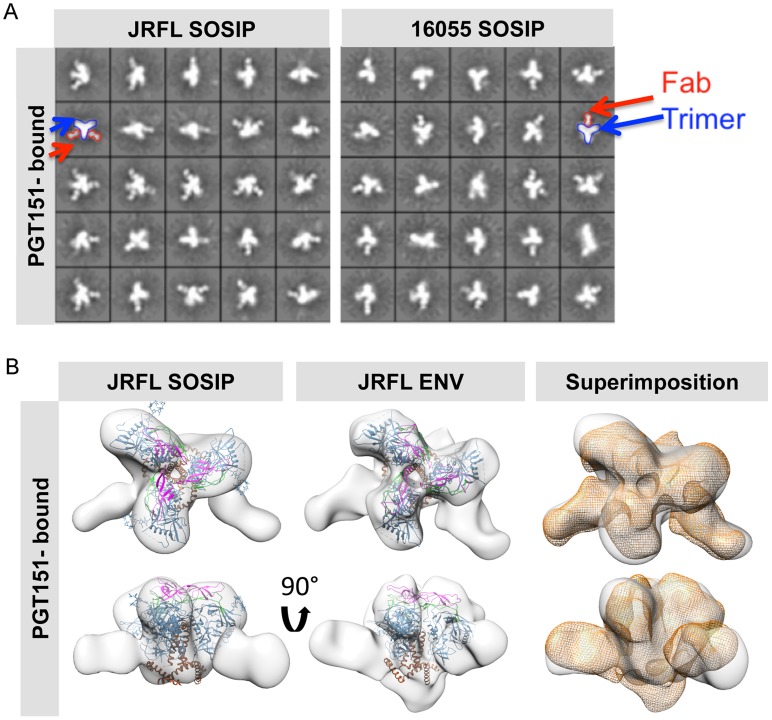
EM 2D class averages and 3D reconstructions of the trimer-preferred cleavage-specific PGT151-bound SOSIP trimers. (A) EM 2D class averages of PGT151-bound JRFL and 16055 SOSIP trimers (left and right, respectively). Red arrows indicate the density corresponding to a PGT151 Fab and the blue arrows indicate the density corresponding to the trimeric proteins. (B) Top and side views of 3D reconstruction EM densities of PGT151-bound JRFL-SOSIP and JRFL-ENV (gray) with the BG505 SOSIP cryo-EM structure (PDB ID 3J5M, gp120 in blue, V1V2 in magenta, V3 in green and gp41 in brown) fitted within. To the right, top and side views of the liganded JRFL SOSIP EM density (gray) superimposed onto the liganded JRFL-ENV EM density (orange).

To solidify that these two SOSIP trimers faithfully mimic the virion native spike conformation, we made complexes with the recently described, trimer-preferring and cleavage-specific bNAb PGT151. This bNAb binds specific N-linked glycans at the interface of four Env subunits, two gp120 and two gp41 protomers [41], [42]. The EM 2D class averages of PGT151 in complex with JRFL and 16055 SOSIP trimers revealed mostly two or one Fabs per trimer (Fig. 7A). Computed stoichiometries based on EM micrographs revealed that ∼36% of the JRFL SOSIP trimers possessed two Fabs, ∼28% with one bound Fab and ∼11% with 3 bound Fabs (S1 Table). PGT151 displayed similar stoichiometry in its interaction with the native JRFL envelope extracted from the cell membrane, as recently described [41]. The subtype C 16055 SOSIP 2D class averages displayed mostly one PGT151 Fab bound per trimer, although two Fabs were occasionally detected (Fig. 7A) and the computed stoichiometry was slightly different than for JRFL (∼34% with one bound Fab, ∼20% with two Fabs and 0% with three Fabs) (S1 Table). The lower stoichiometry for 16055 is consistent with the observation that in ∼60% of cases, only one PGT151 Fab bound the subtype C C22 Env following extraction from the cell membrane [41]. Based on the more favorable stoichiometry with PGT151 and on the availability of the native JRFL Env-PGT151 complex EM density [41], we obtained a 3D EM model of the JRLF SOSIP bound to PGT151 at 24 Å resolution (Fig. 7B). This 3D model displays two Fabs bound per trimer and its superimposition with that of the JRFL cleaved full-length Env purified in complex with PGT151 showed a high degree of correspondence, with the expected exceptions of the MPER and TM gp41 regions lacking in the SOSIP trimers (Fig. 7B). We also superimposed the JRFL SOSIP-PGT151 3D reconstruction with that of the published BG505 SOSIP-PGT151 density and, as expected, they were highly concordant (S6A Fig.).

### Different levels of stability are displayed by the JRFL and 16055 SOSIP trimers as assessed by biophysical measurements

**Figure 8:**
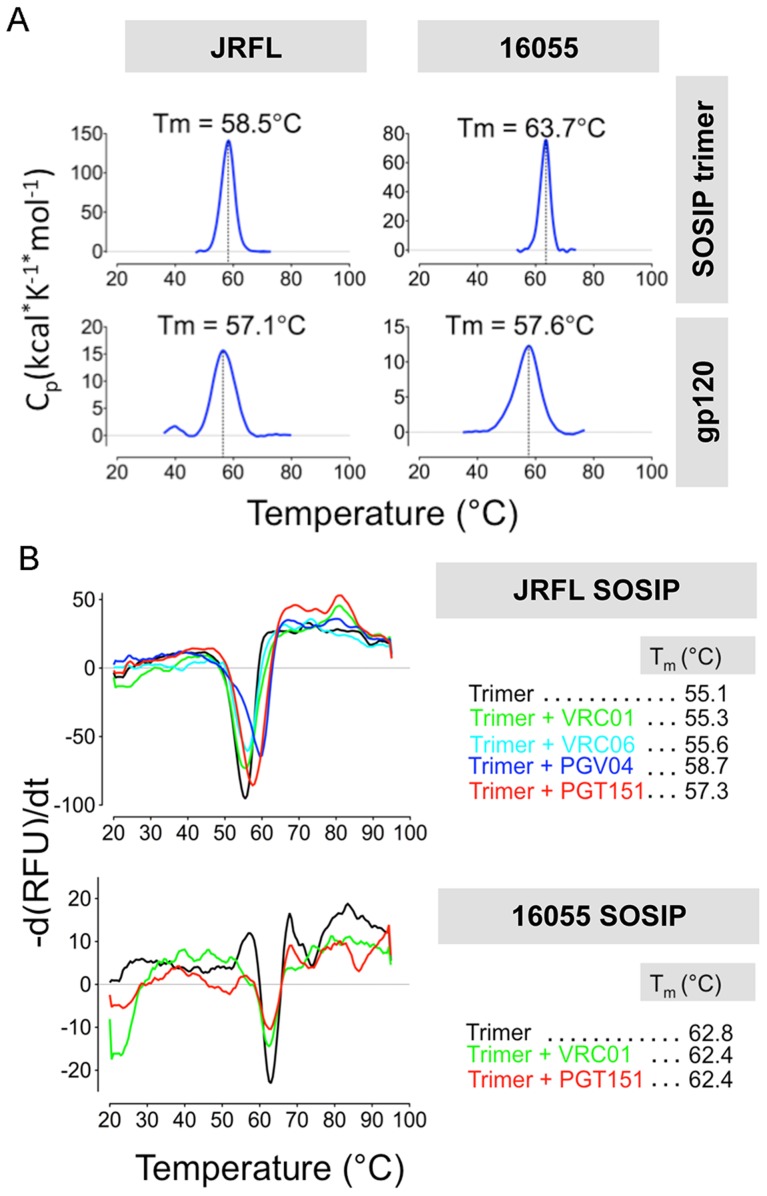
Analysis of SOSIP trimer thermal stability by DSC and DSF. (A) DSC analysis of the negatively selected JRFL and 16055 SOSIP trimers and its gp120 monomer controls. (B) DSF analysis of JRFL and 16055 SOSIP trimers alone and pre-incubated with a 2-fold M excess of selected Fabs.

We assessed the stability of the negatively selected SOSIP trimers by two biophysical methods, differential scanning calorimetry (DSC) and differential scanning fluorimetry (DSF). By DSC, the JRFL SOSIP trimer displayed a melting temperature (T_m_) of 58.5°C, about 1°C higher than the JRFL gp120 monomer T_m_ of 57.1°C. In contrast, the 16055 SOSIP trimer melted at 63.7°C, approximately 6 degrees higher than the 16055 gp120 monomer (57.6°C) (Fig. 8A). Just as some mAbs can induce conformational changes on the trimer, we reasoned that some might also stabilize the trimeric ground-state. Accordingly, we used DSF to measure melting temperatures in the context of liganded-SOSIP trimers, a comparable method to DSC that requires less protein and is more amenable to higher throughput analysis. DSF employs a real-time PCR instrument to detect fluorescence emission of a dye with specificity for hydrophobic residues. The exposure of hydrophobic residues as the protein unfolds with increasing temperature/energy results in a sigmoidal curve that allows the determination of the protein melting temperature. By DSF, the melting temperatures of the JRFL and 16055 SOSIP trimers alone were 55.1°C and 62.8°C, respectively, comparable to those determined by DSC (Fig. 8B). We selected the CD4bs-directed bNAbs (VRC01, PGV04 and VRC06) and the trimer-preferring mAb (PGT151) to investigate their stabilizing or destabilizing effect on the SOSIP trimers. While VRC01 and VRC06 had no significant effect in trimer stability, the antibodies PGT151 and PGV04 increased the T_m_ of the JRFL SOSIP-mAb complex by 2.1 and 3.6°C, respectively (Fig. 8B). The melting temperatures of the individual Fabs were plotted separately for clarity (S6C Fig.). These data may be of value for subsequent immunogenicity assessments using immune complexes to enhance SOSIP stability *in vivo*. In contrast, 16055 SOSIP trimers in complex with either VRC01 or PGT151 mAbs did not increase the T_m_ beyond that of the trimer alone (Fig. 8B).

In addition, to further assess trimer stability by EM, we compared the 3D models of JRFL SOSIP bound to the PGV04 Fabs at days 0 and 7 following “trimer alone” incubation at 4°C. We observed that the trimeric complexes appeared similar at both time points, indicating no deterioration in quaternary structure over this time interval (S7 Fig.).

## Discussion

In this study, we selected two HIV-1 Env sequences from subtypes B and C to produce soluble SOSIP trimers to complement the already available subtype A BG505 SOSIP trimer. Obtaining soluble mimetics of the native HIV-1 spike from subtypes representing the majority of global infections is of high interest for additional structural analysis as well as preclinical immunogenicity studies and candidate vaccine trials. The JRFL and 16055 SOSIPs did form well-ordered trimers, but not as readily as the subtype A SOSIP trimers derived from the BG505 Env sequences [24], [30], indicating molecular heterogeneity. Since, following SEC, we detected well-ordered SOSIP trimers within a mixture of Env forms, we used negative selection to purify the JRFL and 16055 SOSIP trimers to a high level of homogeneity. We then were able to obtain 3D EM reconstructions of these trimers in both the unliganded state and in complex with sCD4 and selected bNAbs. By EM, we demonstrated that sCD4 induced conformational changes in these SOSIP trimers that parallel those observed for the native BaL-Env spike. In addition, the cleavage- and trimer-specific bNAb PGT151 recognized the JRFL SOSIP trimers in a manner similar to its recognition of the native JRFL Env spike. We demonstrated that the antigenic profile of the negatively selected trimers was consistent with a well-ordered state, mimicking the viral spike and that the trimers exhibited degrees of thermostability consistent with a homogenous species by calorimetry and fluorimetry.

To obtain the well-ordered trimers, we used CD4bs-directed non-bNAbs to selectively adsorb the disordered oligomers to the solid phase. F105 readily removed the disordered oligomers from the JRFL SOSIP mixture but was insufficient for 16055, likely due to its faster off-rate for the 16055 gp120 monomer. GE136 was a better negative selecting agent for the 16055 mixture showing a considerably slower dissociation rate for monomeric 16055 gp120 (S1B Fig. and S3 Fig.). A slower dissociation rate of a given mAb for the gp120 monomer is likely advantageous to efficiently capture the undesirable disordered oligomers within the SOSIP mixture and may be a key factor for successful negative selection.

Following negative selection, we isolated highly homogeneous well-ordered JRFL and 16055 SOSIP trimers, which share conformational and antigenic similarities with the BG505 SOSIP.664 trimers. BG505 SOSIP.664 do not require this negative-selection purification step since, following initial 2G12 positive selection, they form well-ordered trimers that can be isolated by SEC alone [30]. Why most HIV-1 Env sequences do not form SOSIP trimers with this degree of homogeneity is not yet clear but seems to be a relatively infrequent feature associated with the BG505 Env, perhaps by specific structural interaction focused around the I559P change that alters gp41 conformational flexibility to inhibit efficient six-helix bundle formation, the post-fusiogenic form of gp41.

The negatively selected JRFL and 16055 SOSIP trimers displayed efficient recognition by bNAbs, including those recognizing quaternary epitopes, and low or undetectable reactivity to CD4bs-targeting non-bNAbs and other non-neutralizing antibodies targeting other sites than the CD4bs, such as 17b and C11. These data suggest that the CD4bs-directed negative-selection process eliminated generally disordered trimers and was not specifically restricted to the CD4bs. Generally, most bNAbs that efficiently recognized the negatively selected trimers, also neutralized the parental sequence viral strain, suggesting that these spike soluble mimetics faithfully recapitulate the quaternary packing of the native Env spike. This interpretation is consistent with the reported correlation between bNAb HIV-1 neutralizing activity and binding to the ordered BG505 SOSIP.664 trimers [30].

Similar to that study, and as reported here, the exception was the V3-targeting antibody 19b that does not neutralize the parental JRFL or 16055 viruses, but did recognize the soluble SOSIP trimers. The binding of 19b was slightly reduced after negative selection, but not fully abrogated, suggesting that the V3 might be in a more exposed or “triggered” conformation at least in one of the protomers within some of the population of trimers. Consistent with this, EM negative stain using 19b Fab with negatively selected JRFL and 16055 SOSIP detected a low percentage of Fab binding and exclusively to one protomer. A similar level of V3 reactivity was also observed previously for BG505 SOSIP.664. This undesirable reactivity might be overcome with additional design modifications [30]. This V3 exposure that might be due to “breathing” of this region in the relatively well-ordered trimeric context, may reflect the metastable condition of the Env spike itself since activation of the HIV-1 spike occurs by protein:protein interaction. It may be that a low energy barrier is required to trigger HIV-1 Env by this means, making conformational breathing more likely. HIV-1 Env is not triggered by pH as is, for example, influenza HA or other endosomally triggered viral fusion units, which may allow a wider degree of trimer stability in the native Env state.

In one other exception, the N332-lacking 16055 SOSIP trimers were recognized by the N332-targeting bNAbs 2G12 and PGT121. 2G12 does not neutralize the parental virus while PGT121 neutralizes the parental virus strain, which is likely related to, and consistent with, the promiscuity of “nearby” glycan usage displayed by this bNAb that does not absolutely require the presence of the 332 N-glycan [45].

Both JRFL and 16055 SOSIP trimers are relatively stable, displaying melting temperatures of 58.5°C and 63.7°C, respectively. These values are substantially in excess of perhaps more relevant temperatures such as room or physiological body temperature. Since the published melting temperature of BG505 SOSIP.664 trimer is higher (68°C) [30], one could postulate that the T_m_ of the SOSIP trimer will correlate with a higher degree of well-ordered trimer formation. However, the melting temperature of 16055 SOSIP was almost 6°C higher than that of JRFL SOSIP and 16055 had a lower percentage of “spontaneous” trimer formation as revealed by the SEC profiling and related analysis. Other factors such as the level of glycosylation or the propensity of the V1/V2/V3 to adopt a near native arrangement even within a protomer may contribute to the higher thermostability of the 16055 or BG505 SOSIP trimers. BG505 and 16055 gp120s, but not JRFL gp120, are weakly recognized by the V2-directed, trimer-preferring antibodies PG9 and PG16. BG505 SOSIP and 16055 SOSIP display higher T_m_s than does JRFL SOSIP, suggesting that the propensity of the 16055 and BG505 monomers to adopt a native, trimer-like conformation may contribute to the thermostability of the SOSIP oligomers.

Antibodies PGV04 and PGT151 increased the T_m_ of the JRFL SOSIP trimer. This could be a result of specific inter-protomer contacts established by the paratope of the antibody, simultaneously bridging two protomers and conferring structural rigidity to the architecture of the trimer. In contrast, VRC01 shows a lower level of this putative inter-protomer bridging as suggested indirectly in the recent publication of the EM structure of the BG505 SOSIP trimer bound to PGV04 [28]. VRC01 did not show a significant stabilization effect on the SOSIP trimers as measured by DSF, and even, destabilized the 16055 SOSIP trimer as evidenced in the EM 3D reconstruction of the complex.

Obtaining soluble mimics of the HIV-1 spike representative of different subtypes/strains of HIV will be of benefit toward potential advancement of a global HIV-1 vaccine. The recently published structural characterization of the subtype A BG505 SOSIP.664 [28], [29], a soluble mimetic of the HIV native spike, provides fundamental insights regarding the organization of the gp120 and gp41 within the trimer. Extending this structural information to other subtype strains of HIV is of high interest in the field. For now, SOSIP represents the best and only well-ordered soluble trimer mimetic. Frustratingly, many HIV sequences do not readily form ordered homogeneous SOSIP trimers to the extent that BG505 Env does, generating different frequencies of well-ordered versus disordered trimers even when they are genetically designed in an identical manner. This trend toward trimer micro-heterogeneity likely explains why, in part, previous attempts to obtain high-resolution crystal structures of the JRFL and KNH1144 SOSIP trimers were not fruitful until BG505 was identified. Our trimer isolation strategy leads to a high-degree of conformational homogeneity that may allow the determination of higher resolution structures of SOSIP trimers derived from other HIV subtypes. In summary, in this study, we offer a new means to obtain homogenous well-ordered SOSIP trimers of subtypes B and C that potentially can be extended to more HIV-1 Env strains by the use of the non-bNAb negative-selection strategy used to rescue well-ordered trimer sub-fractions of JRFL and 16055 SOSIP oligomers.

## Materials and Methods

### Design of the JRFL SOSIP and 16055 SOSIP constructs

To generate the HIV-1 subtypes B JRFL SOSIP and C 16055 SOSIP expression plasmids we generally followed established SOSIP design parameters [30]. In brief, the JRFL and 16055 SOSIP trimers were engineered with a disulfide linkage between gp120 and gp41 (residue 501 in gp120 to residue 605 in gp41) that covalently links the two subunits of the heterodimer (SOS) [33]. As previously described, we included the I559P mutation in the heptad repeat region 1 (HR1) of gp41 that promotes trimerization of the heterodimer, and a deletion of part of the hydrophobic membrane proximal external region (MPER), in this case residues 664–681 of the Env ectodomain [31], [34], [35]. The furin cleavage site between gp120 and gp41 (_508_REKR_511_) was altered (_506_RRRKKR_511_) to enhance cleavage [47]. The JRFL SOSIP trimer includes an additional mutation (E168K) that is associated with PG9/PG16 neutralization sensitivity in the pseudovirus neutralization assay that is naturally present in the 16055 Env [48], [49]. The CD5 leader sequence was positioned at the 5′ end of the SOSIP encoding DNA to enhance secretion and expression.

### Purification of soluble trimers

JRFL SOSIP and 16055 SOSIP expression constructs were transfected into 293F cells along with a plasmid encoding the cellular protease furin to ensure efficient cleavage of the Env precursor gp160 at a 2∶1 Env:furin ratio [50]. The transfected 293F cells were cultured in a CO_2_ humidified shaking incubator at 37°C for 5–6 days to transiently express the soluble SOSIP trimers. Culture supernatants were collected and cells were removed by centrifugation at 3800 x g for 20 min, filtered twice, first with a 0.45 µm pore size filter device (Nalgene) and subsequently with a 0.2 µm pore size filter. SOSIP proteins were purified by flowing the supernatant over a lectin (*Galanthus nivalis*) affinity chromatography column overnight at 4°C. Proteins were eluted from the lectin column with 3 column volumes of 0.5 M methyl-α-D-mannopyranoside and 0.5 M NaCl. The eluate was concentrated with a Millipore concentrator (MWCO 100 kDa) to 500 µL and loaded onto a Superdex 200 10/300 GL column to separate the trimer-size oligomers from aggregates and gp140 monomers. Fractions corresponding to the trimer (approximately eluate volumes 10–12 mL) were pooled and loaded into an agarose protein A column previously loaded with a 1 mg of mAb (F105 or GE136) per ml of column material. This quantity can be customized in relation to the amount of SOSIP protein loaded. Generally, we used 2-fold weight excess of mAb with respect to SOSIP protein amount obtained after SEC. Specifically, we used F105 for JRFL SOSIP and GE136 for 16055 SOSIP trimer purification. The column was rocked at 4°C for 45 min, the solid phase was allowed to settle for 5 min and the flow-through collected by flowing one column volume of PBS through the column. The flow-through, containing the well-ordered trimers, was concentrated using a 100 kDa molecular weight cut off filter device from Millipore to approximately 1.5 mg/mL for analysis or cold storage. Final yields of JRFL SOSIP well-ordered trimers following negative selection were approximately 1.5 mg/L and 0.5 mg/L for the well-ordered 16055 SOSIP trimers.

### Immunoprecipitation

To perform immunoprecipitation experiments on supernatants containing the SOSIP trimers, 20 µL of protein A agarose beads were added to a 1.5 ml Eppendorf tube, washed twice with PBS, resuspended in 500 µL of PBS and 5 µg of antibody were added. The protein A agarose-mAb mixture was rocked for 30 min at 4°C and then washed twice with PBS 500 mM NaCl. One mL of filtered supernatant was added to the microtube and rocked for 30 min at 4°C. The microtube was then centrifuged at 1000×g for 5 min and the supernatant discarded. The protein A-agarose pellets containing the bound antibody-Env protein were washed twice with 1 mL of PBS before resuspending them in 20 µL of SDS-PAGE loading buffer to resolve over SDS PAGE minigels for 50 min at 200 V.

### Bio-Layer interferometry (BLI) binding analysis and kinetics

For the binding experiments shown in Fig. 3, Fig. 4, S2 Fig. and S3 Fig. we used an Octet Red instrument immobilizing IgGs on hydrated (PBS pH 7.4) anti-human IgG Fc sensors (AHC: ForteBio). The SOSIP trimers and gp120 monomers were analyzed as analytes in solution (PBS pH 7.4). Briefly, the bio-sensors were immersed in PBS pH 7.4 containing IgGs at a concentration of 10 ug/mL for 2 min and with vibration at 1000 rpm prior to encounter with the analyte (SOSIP trimers or gp120 monomer, 200 nM and 600 nM respectively). The IgG-immobilized sensor was immersed in the analyte in solution for 3 min at 1000 rpm and then removed from the analyte solution and placed into PBS, pH 7.4, for an additional 3 min. The 3 min binding intervals generated the association and dissociation binding curves reported in this study. For the data reported in Fig. 5A, Fabs and monovalent 2G12 IgG were used as analytes in solution (400 nM–25 nM) and the JRFL SOSIP trimer were immobilized on an anti-His biosensors (HIS2; ForteBio) at a concentration of 10 ug/mL.

### Electron microscopy

Negatively selected JRFL and 16055 SOSIP trimeric proteins were incubated with a ten molar excess of selected Fabs at RT for 1 hour. The following complexes were analyzed 1. JRFL-SOSIP with four-domain sCD4, 2. JRFL-SOSIP with VRC01, 3. JRFL-SOSIP with VRC03, 4. JRFL-SOSIP with PGT151, 5. JRFL-SOSIP with PGV04, 6. 16055 SOSIP with four-domain sCD4, 7. 16055 SOSIP with VRC01, 8. 16055 SOSIP with VRC03, and 9. 16055 SOSIP with PGT151. A 3 µL aliquot containing ∼0.05 mg/ml of the Fab+JRFL-SOSIP complex or the Fab+16055 complex was applied for 15 s onto a carbon coated 400 Cu mesh grid that had been glow discharged at 20 mA for 30 s, followed by negative staining with 2% uranyl formate for 30 s. Data were collected using a FEI Tecnai Spirit electron microscope operating at 120 keV, with an electron dose of ∼36 e^−^/Å^2^ and a magnification of 52,000x that resulted in a pixel size of 2.05 Å at the specimen plane. Images were acquired with a Tietz 4 k×4 k TemCam-F416 CMOS camera using a nominal defocus of 1000 nm and the Leginon package at 10° tilt increments, up to 50°. The tilts provided additional particle orientations to improve the image reconstructions.

### Data processing and image reconstruction

Particles were picked automatically using DoG Picker and put into a particle stack using the Appion software package [51], [52]. Initial, reference-free, two-dimensional (2D) class averages were calculated using particles binned by two via Xmipp Clustering 2D Alignment [53] and sorted into classes. Particles corresponding to complexes were selected into a substack and binned by two before another round of reference-free alignment was carried out using the Xmipp Clustering and 2D alignment and IMAGIC programs [54]. Fabs and sCD4 were clearly visualized in the 2D class averages if they are bound to the trimer, allowing the percentage of bound trimers relative to unbound trimers to be tabulated (S1 Table) [28]. *ab initio* common lines models were calculated from reference-free 2D class averages in EMAN2 [55] without imposing symmetry. All *ab initio* common lines models were the same. One of those models was then refined against raw particles for an additional 89 cycles. EMAN [56] was used for all 3D reconstructions. The resolutions of the final models were determined using a Fourier Shell Correlation (FSC) cut-off of 0.5 (S6B Fig. and S8 Fig.). The number of particles used for the 3D reconstructions are shown in S1 Table.

### Model fitting into the EM reconstructions

The cryo-EM structure of PGV04-liganded BG505 SOSIP.664 (3J5M), and gp120 with sCD4 (1RZK) were manually fitted into the EM densities and refined by using the UCSF Chimera [57] ‘Fit in map’ function.

### Differential Scanning Calorimetry (DSC)

Thermal denaturation was analyzed with a N-DSC II differential scanning calorimeter from Calorimety Sciences Corp. (Prov, UT), at a scanning rate of 1 K/min under 3.0 atmospheres of pressure. Samples were dialyzed in PBS pH 7.4 and protein concentration was adjusted to 0.5 mg/mL prior to measurement. Data collected were analyzed after buffer correction, normalization and baseline subtraction.

### Differential Scanning Fluorimetry (DSF)

Fluorescence measurements were performed using a CFX96 RT-PCR detection system (BIO-RAD, Hercules, CA). SYPRO Orange dye was diluted 1∶5000 in PBS pH 7.4 and added to 30 µg of protein in clear PCR tubes to a final volume of 25 µL. For samples containing trimer and Fab complexes, 30 ug of trimer protein were mixed with 10 ug of Fab and incubated at 4°C for 1 hr prior to adding the dye. The fluorescence emission was collected using a fluorescence resonance energy transfer filter (560–580 nm) and an excitation wavelength of 450–490 nm. During the DSF experiment, the temperature was increased from 20 to 95°C at an increment of 0.5°C with an equilibration time of 5 s at each temperature prior to measurement. The data were exported into CFX Manager version 1.6 for analysis. The melting temperature (T_m_) is defined as the temperature corresponding to the minimum value of the negative first derivative of the first fluorescence transition. We note that the high initial fluorescence is likely due to exposure of hydrophobic pockets on the surface of the trimer.

## Supporting Information

S1 Fig **SOSIP design template and SDS-PAGE analysis of JRFL- and 16055-derived proteins.** (A) Schematic representation of the JRFL and 16055 SOSIP linear organization and design modifications. (B) SDS-PAGE gel shows bands corresponding to immuno-precipitated g120 subunit and the heavy and light chain of the mAb from 1 mL of cell culture supernatant containing overexpressed JRFL or 16055 SOSIP glycoproteins (left). Binding kinetic constants of non-bNAbs F105, GE136 and GE148 to monomeric JRFL and 16055 gp120 (right). (C) SDS-PAGE gel analyzing both negatively selected JRFL and 16055 SOSIP trimers with and without DTT. A strong band corresponding to gp120 and a faint band corresponding to the gp41 ectodomain migrated at the expected molecular weight (MW) in the presence of the reducing reagent (DTT). A higher MW band consistent with a disulfide-linked gp120-gp41 is observed in the absence of DTT. Uncleaved JRFL gp140-Foldon (FD) trimers and corresponding JRFL and 16055 gp120 monomers are shown as controls.
(TIF)

S2 Fig **Bio-layer light interferometry (BLI) analysis of JRFL SOSIP trimers and gp120 monomers.** (A) A panel of HIV-1 mAb IgGs were immobilized on anti-human IgG Fc sensors. JRFL SOSIP (200 nM) before and after negative selection and monomeric JRFL gp120 (600 nM) were assessed as analytes in solution (PBS, pH 7.4) to generate the BLI curves. CD4-IgG was used to estimate the concentration of gp120 that would give a similar magnitude response relative to SOSIP trimeric protein. Black curves depict binding events between monomeric gp120 in solution and the corresponding immobilized ligand. The red and blue curves depict binding parameters of the SOSIP trimeric proteins before and after negative selection. The association and dissociation phases were 180 s each in duration. (B) Bars represent BLI maximal responses derived from the curves shown above corresponding to the binding analysis of the negatively selected JRFL SOSIP trimers by bNAbs (blue) and non-bNAbs (red).
(TIF)

S3 Fig **BLI analysis of 16055 SOSIP trimers and gp120 monomers.** (A) A panel of HIV-1 mAb IgGs were immobilized on anti-human IgG Fc sensors. 16055 SOSIP (200 nM) before and after negative selection and monomeric 16055 gp120 (600 nM) were assessed as analytes in solution (PBS, pH 7.4) to generate the BLI curves. CD4-IgG was used to estimate the concentration of gp120 that would give a similar magnitude response relative to SOSIP trimeric protein. Black curves depict binding events between monomeric gp120 in solution and the corresponding immobilized ligand. The red and blue curves depict binding parameters of the SOSIP trimeric proteins before and after negative selection. The association and dissociation phases were 180 s each in duration. (B) Bars represent BLI maximal responses derived from the curves shown above corresponding to the binding analysis of the negatively selected 16055 SOSIP trimers by bNAbs (blue) and non-bNAbs (red).
(TIF)

S4 Fig **EM 2D class averages of 19b-bound SOSIP trimers and comparison of sCD4-bound trimer 3D EM models.** (A) Negatively selected JRFL SOSIP and 16055 SOSIP trimers incubated with the V3-directed non-bNAb, 19b. The blue arrow indicates a trimer and the red arrow indicates a Fab. (B) Superimposition of four-domain sCD4-bound JRFL SOSIP (left) and 16055 SOSIP (middle) in gray over the two-domain sCD4-bound KNH1144 SOSIP.664 in orange (EMD 5708). For comparison, on the right is the two-domain sCD4-liganded native BaL Env (EMD 5455).
(TIF)

S5 Fig **Thermodynamic measurements for sCD4- and VRC01- liganded SOSIP trimers.** Panels depict raw data corresponding to the interaction of four-domain sCD4 (left) and VRC01 Fab (right) with JRFL SOSIP (top) and 16055 SOSIP (bottom). Below each panel the thermodynamic parameters for each measurement are displayed.
(TIF)

S6 Fig **Comparative 3D EM models of PGT151-bound SOSIP and T_m_ determination of Fabs by DSF.** (A) PGT151-bound JRFL SOSIP and BG505-SOSIP.664 (EMD 5921). (B) PGT151-bound JRFL SOSIP projection matching and Fourier Shell correlation graph. (C) Differential scanning fluorimetric (DSF) measurements of Fabs (30 ug).
(TIF)

S7 Fig **EM 3D reconstructions of PGV04-liganded JRFL SOSIP trimer before and after a 7 day incubation.** Top and side views of the EM 3D reconstruction densities of the PGV04-liganded JRFL SOSIP trimer at day 0 (left) and at day 7 (middle) after incubation at 4°C. JRFL SOSIP in gray with the high resolution cryo-EM structure of the PGV04-liganded BG505 SOSIP.664 (PDB 3J5M, gp120 in blue with V1V2 in magenta, V2 in green, gp41 in brown and the PGV04 Fab in red) fitted within. Top and side views of the liganded JRFL SOSIP at 7 days (orange) superimposed onto the liganded JRFL SOSIP at day 0 (gray).
(TIF)

S8 Fig **Projection matching and Fourier Shell correlation graphs.** (A) Un-liganded (left) and sCD4-bound (right) 16055 and JRFL SOSIP (B) VRC01-bound (left) and VRC03-bound (right) 16055 and JRFL SOSIP.
(TIF)

S1 Table **Antibody neutralization of HIV-1 JRFL and 16055 and stoichiometry of selected Fabs on SOSIP trimers by EM.** Antibody neutralization of HIV-1 JRFL and 16055 viral strains (Top). Fab occupancy detected by EM on JRFL and 16055 SOSIP trimers by EM (Middle). Number of particles computed for the determination of 3D EM reconstructions of JRFL and 16055 SOSIP trimers with selected Fabs.
(DOCX)
