# Supplementary figures and images for: Well-Ordered Trimeric HIV-1 Subtype B and C Soluble Spike Mimetics Generated by Negative Selection Display Native-like Properties
Source: PLoS Pathog. 2015 Jan 8;11(1):e1004570. doi: 10.1371/journal.ppat.1004570 (PMC4287557; doi:10.1371/journal.ppat.1004570)

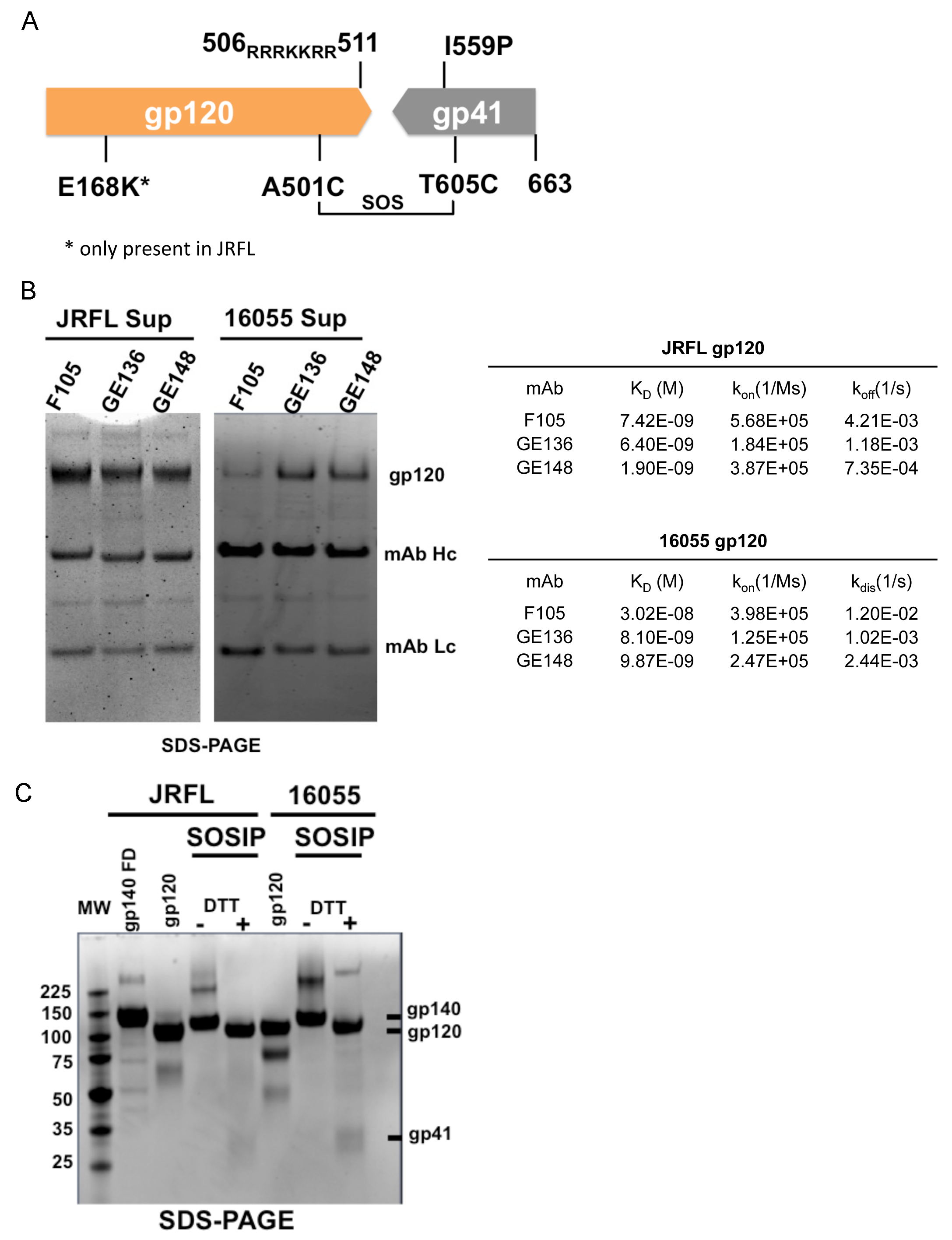

Supplement: S1 Fig — SOSIP design template and SDS-PAGE analysis of JRFL- and 16055-derived proteins. (A) Schematic representation of the JRFL and 16055 SOSIP linear organization and design modifications. (B) SDS-PAGE gel shows bands corresponding to immuno-precipitated g120 subunit and the heavy and light chain of the mAb from 1 mL of cell culture supernatant containing overexpressed JRFL or 16055 SOSIP glycoproteins (left). Binding kinetic constants of non-bNAbs F105, GE136 and GE148 to monomeric JRFL and 16055 gp120 (right). (C) SDS-PAGE gel analyzing both negatively selected JRFL and 16055 SOSIP trimers with and without DTT. A strong band corresponding to gp120 and a faint band corresponding to the gp41 ectodomain migrated at the expected molecular weight (MW) in the presence of the reducing reagent (DTT). A higher MW band consistent with a disulfide-linked gp120-gp41 is observed in the absence of DTT. Uncleaved JRFL gp140-Foldon (FD) trimers and corresponding JRFL and 16055 gp120 monomers are shown as controls. (TIF) [file ppat.1004570.s001.tif]

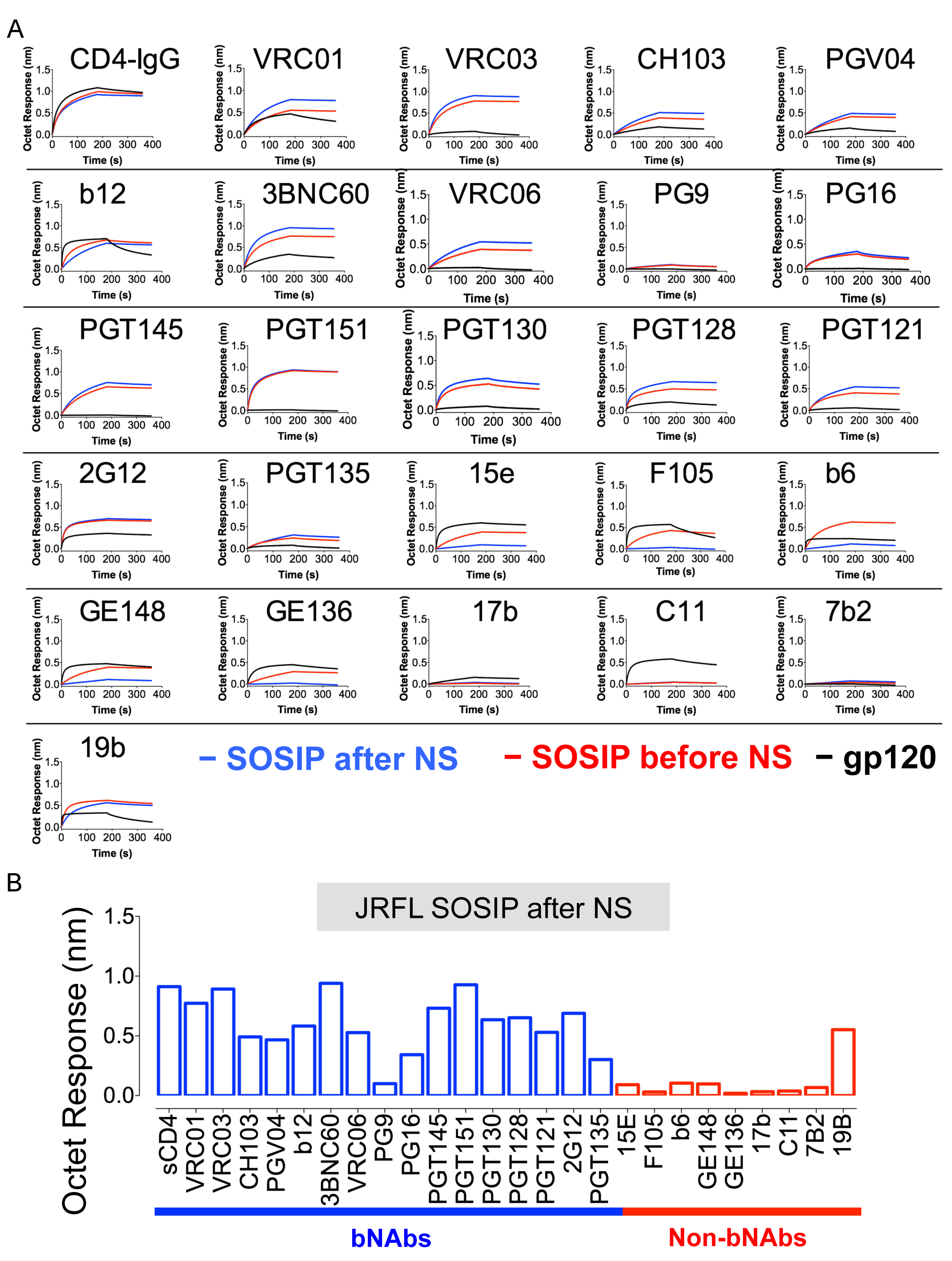

Supplement: S2 Fig — Bio-layer light interferometry (BLI) analysis of JRFL SOSIP trimers and gp120 monomers. (A) A panel of HIV-1 mAb IgGs were immobilized on anti-human IgG Fc sensors. JRFL SOSIP (200 nM) before and after negative selection and monomeric JRFL gp120 (600 nM) were assessed as analytes in solution (PBS, pH 7.4) to generate the BLI curves. CD4-IgG was used to estimate the concentration of gp120 that would give a similar magnitude response relative to SOSIP trimeric protein. Black curves depict binding events between monomeric gp120 in solution and the corresponding immobilized ligand. The red and blue curves depict binding parameters of the SOSIP trimeric proteins before and after negative selection. The association and dissociation phases were 180 s each in duration. (B) Bars represent BLI maximal responses derived from the curves shown above corresponding to the binding analysis of the negatively selected JRFL SOSIP trimers by bNAbs (blue) and non-bNAbs (red). (TIF) [file ppat.1004570.s002.tif]

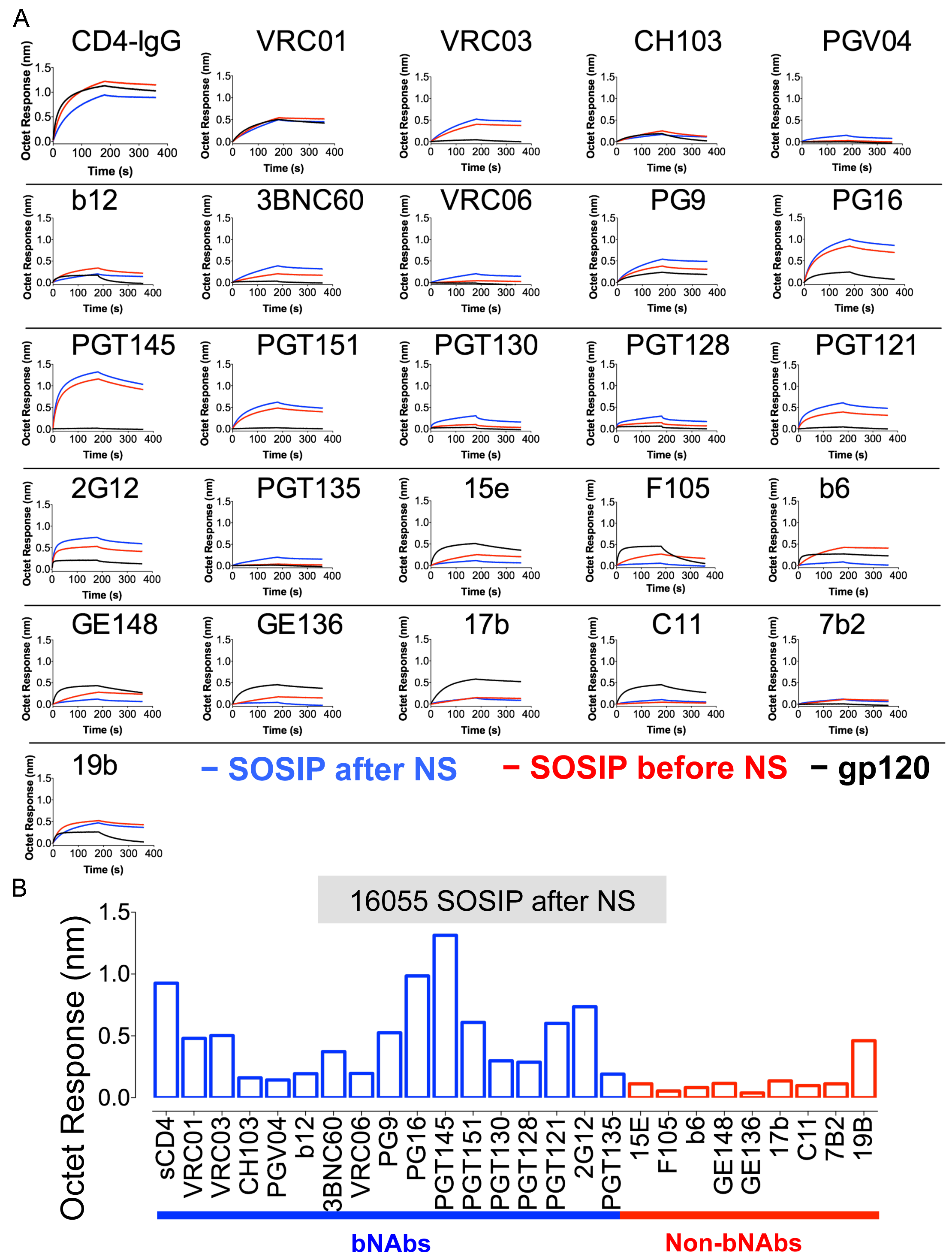

Supplement: S3 Fig — BLI analysis of 16055 SOSIP trimers and gp120 monomers. (A) A panel of HIV-1 mAb IgGs were immobilized on anti-human IgG Fc sensors. 16055 SOSIP (200 nM) before and after negative selection and monomeric 16055 gp120 (600 nM) were assessed as analytes in solution (PBS, pH 7.4) to generate the BLI curves. CD4-IgG was used to estimate the concentration of gp120 that would give a similar magnitude response relative to SOSIP trimeric protein. Black curves depict binding events between monomeric gp120 in solution and the corresponding immobilized ligand. The red and blue curves depict binding parameters of the SOSIP trimeric proteins before and after negative selection. The association and dissociation phases were 180 s each in duration. (B) Bars represent BLI maximal responses derived from the curves shown above corresponding to the binding analysis of the negatively selected 16055 SOSIP trimers by bNAbs (blue) and non-bNAbs (red). (TIF) [file ppat.1004570.s003.tif]

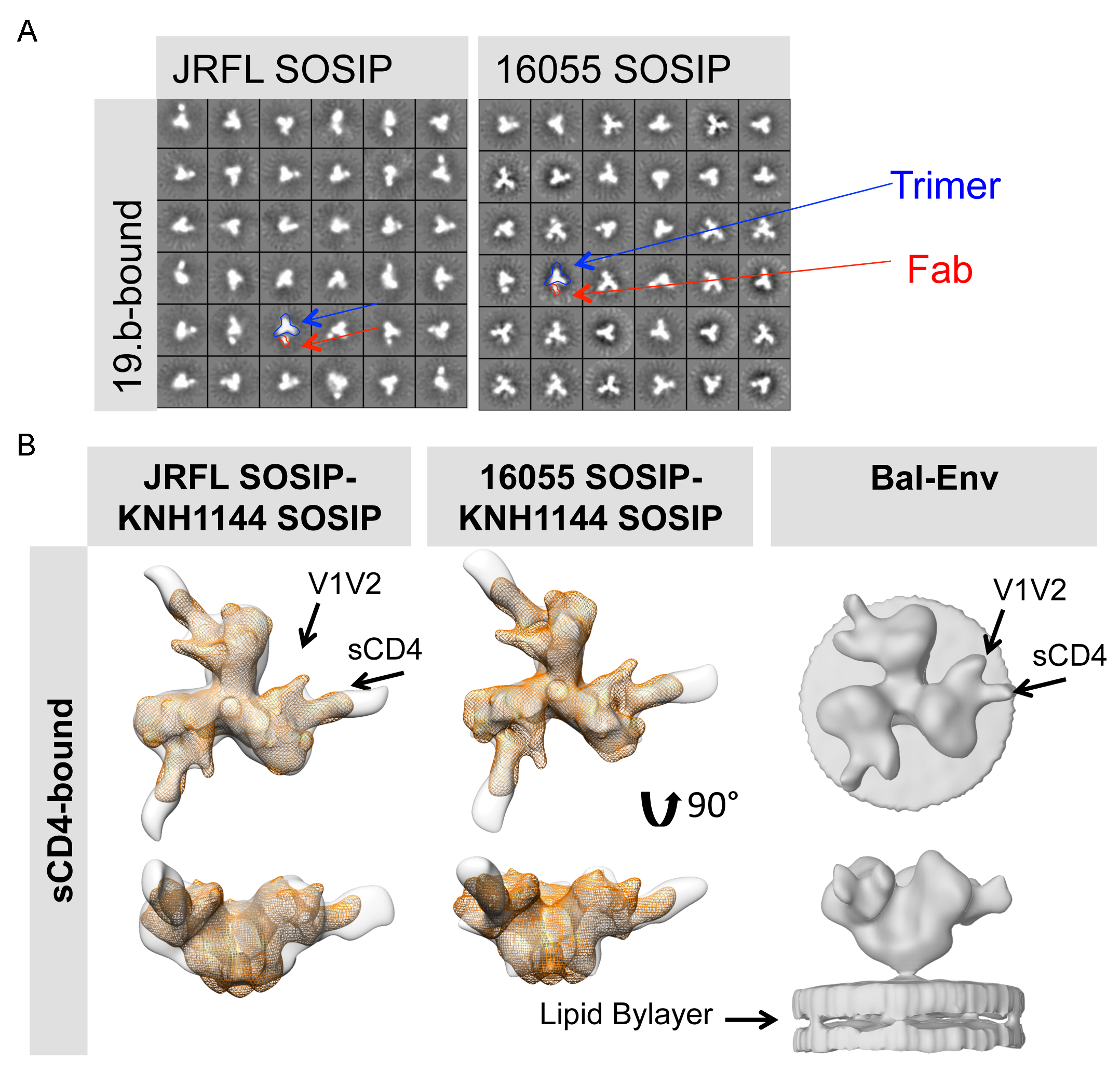

Supplement: S4 Fig — EM 2D class averages of 19b-bound SOSIP trimers and comparison of sCD4-bound trimer 3D EM models. (A) Negatively selected JRFL SOSIP and 16055 SOSIP trimers incubated with the V3-directed non-bNAb, 19b. The blue arrow indicates a trimer and the red arrow indicates a Fab. (B) Superimposition of four-domain sCD4-bound JRFL SOSIP (left) and 16055 SOSIP (middle) in gray over the two-domain sCD4-bound KNH1144 SOSIP.664 in orange (EMD 5708). For comparison, on the right is the two-domain sCD4-liganded native BaL Env (EMD 5455). (TIF) [file ppat.1004570.s004.tif]

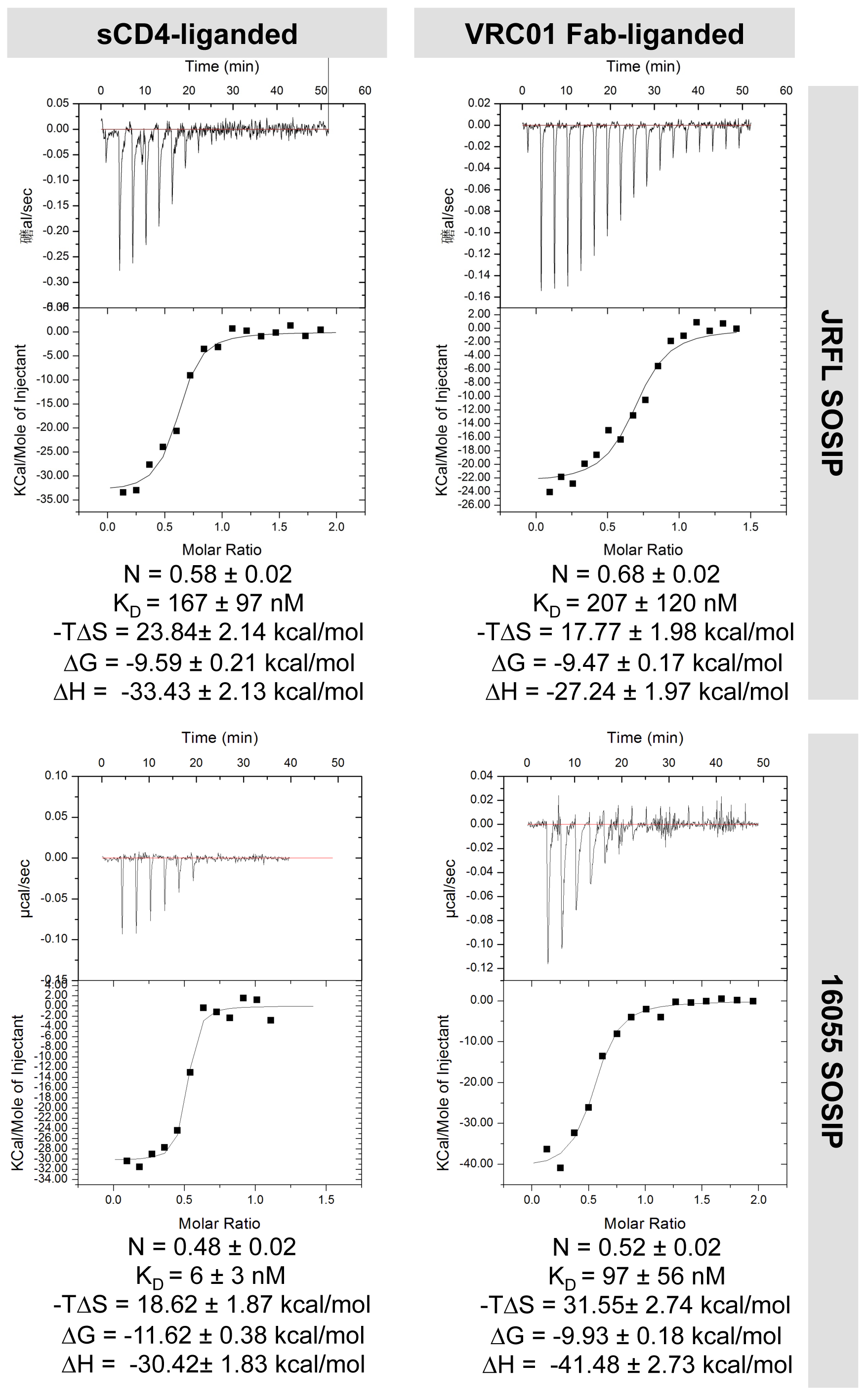

Supplement: S5 Fig — Thermodynamic measurements for sCD4- and VRC01- liganded SOSIP trimers. Panels depict raw data corresponding to the interaction of four-domain sCD4 (left) and VRC01 Fab (right) with JRFL SOSIP (top) and 16055 SOSIP (bottom). Below each panel the thermodynamic parameters for each measurement are displayed. (TIF) [file ppat.1004570.s005.tif]

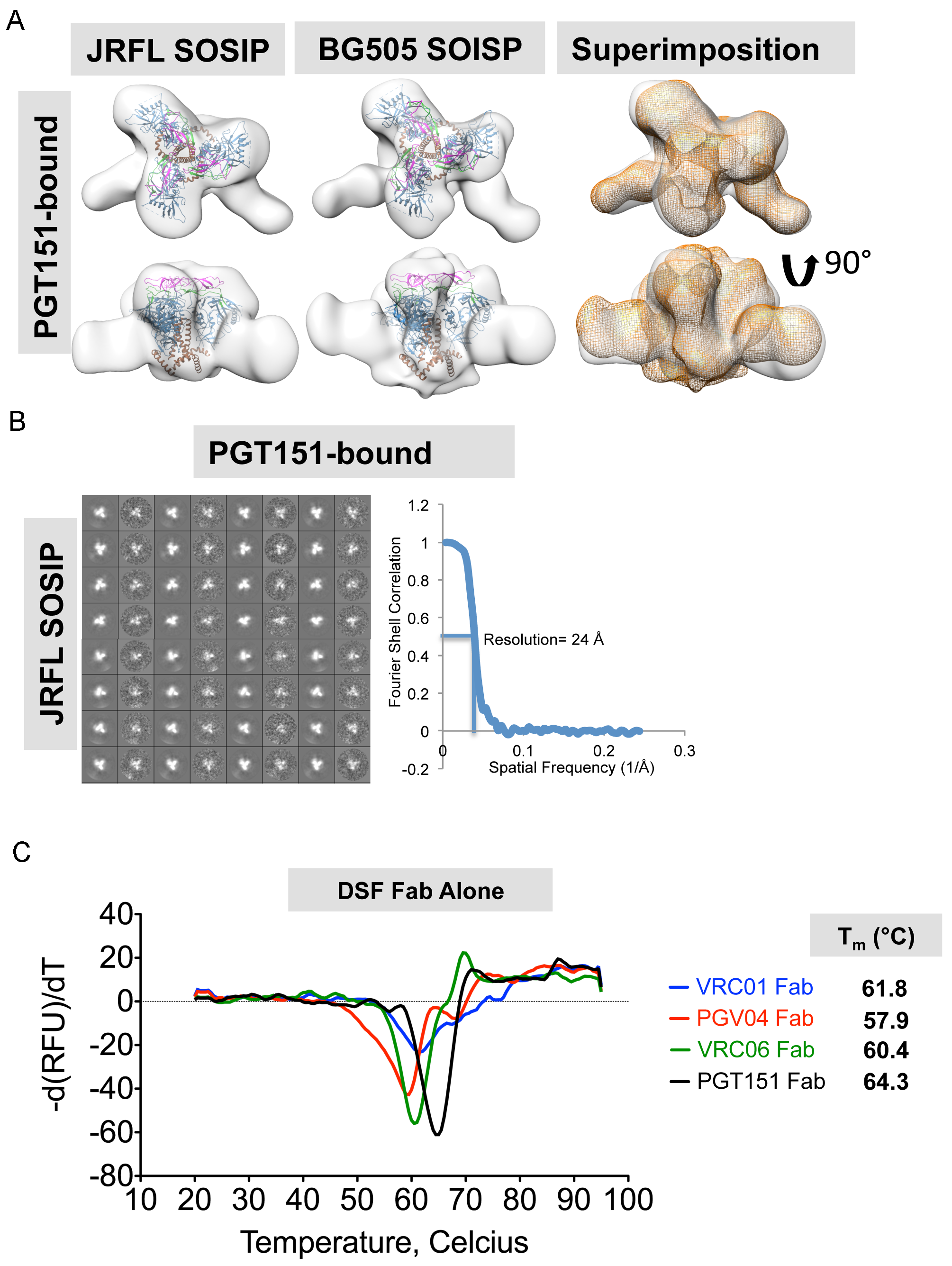

Supplement: S6 Fig — Comparative 3D EM models of PGT151-bound SOSIP and Tm determination of Fabs by DSF. (A) PGT151-bound JRFL SOSIP and BG505-SOSIP.664 (EMD 5921). (B) PGT151-bound JRFL SOSIP projection matching and Fourier Shell correlation graph. (C) Differential scanning fluorimetric (DSF) measurements of Fabs (30 ug). (TIF) [file ppat.1004570.s006.tif]

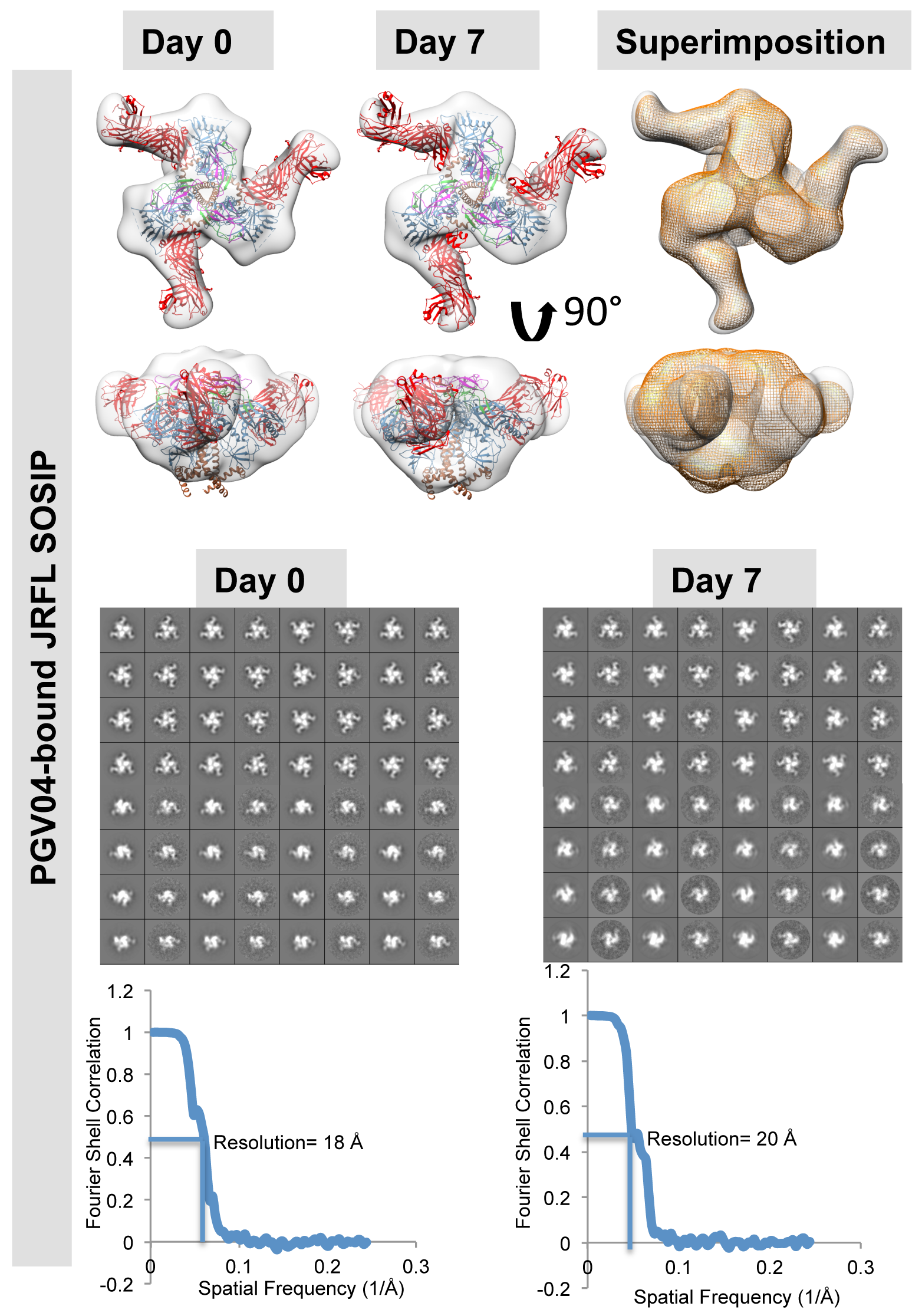

Supplement: S7 Fig — EM 3D reconstructions of PGV04-liganded JRFL SOSIP trimer before and after a 7 day incubation. Top and side views of the EM 3D reconstruction densities of the PGV04-liganded JRFL SOSIP trimer at day 0 (left) and at day 7 (middle) after incubation at 4°C. JRFL SOSIP in gray with the high resolution cryo-EM structure of the PGV04-liganded BG505 SOSIP.664 (PDB 3J5M, gp120 in blue with V1V2 in magenta, V2 in green, gp41 in brown and the PGV04 Fab in red) fitted within. Top and side views of the liganded JRFL SOSIP at 7 days (orange) superimposed onto the liganded JRFL SOSIP at day 0 (gray). (TIF) [file ppat.1004570.s007.tif]

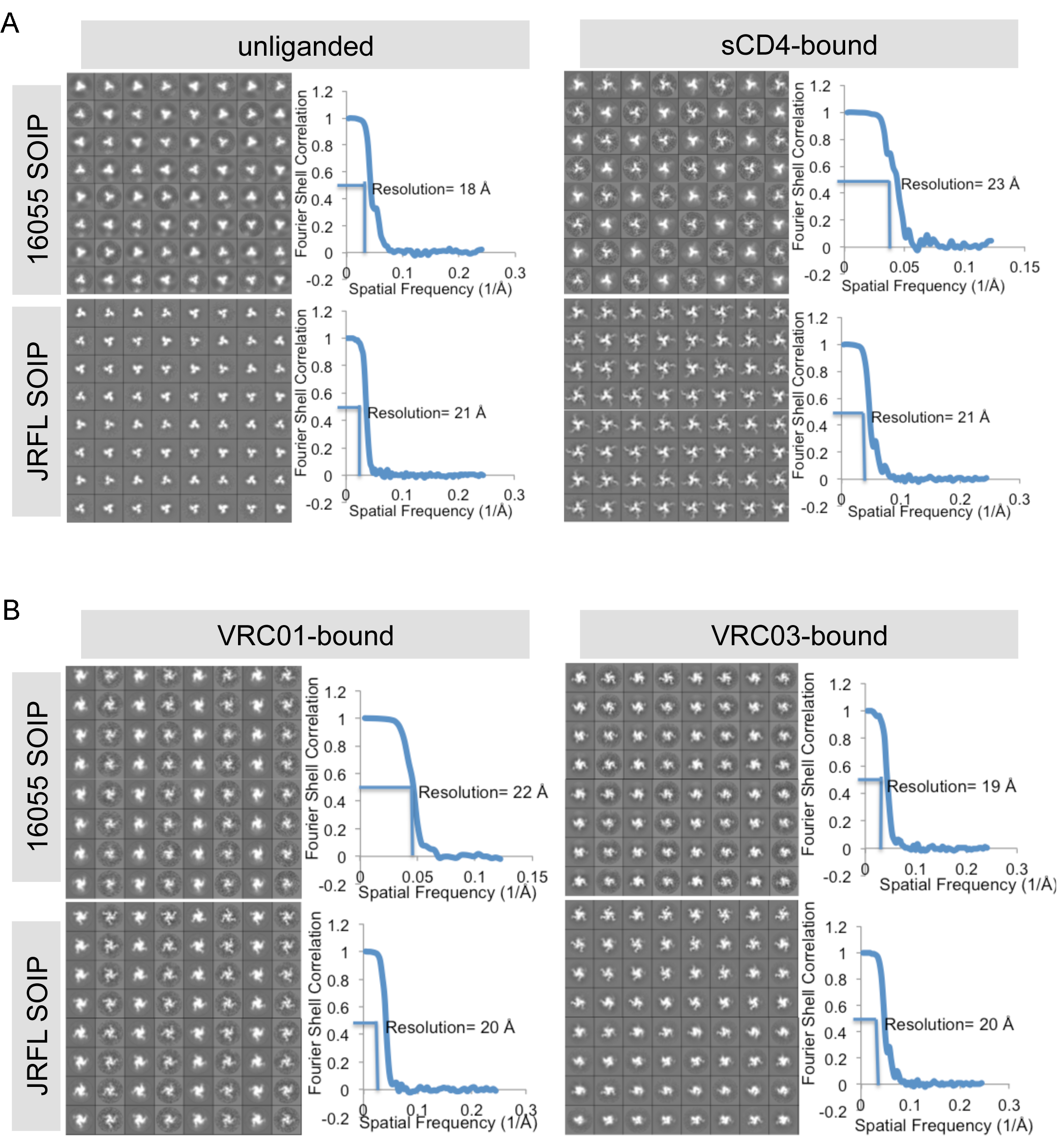

Supplement: S8 Fig — Projection matching and Fourier Shell correlation graphs. (A) Un-liganded (left) and sCD4-bound (right) 16055 and JRFL SOSIP (B) VRC01-bound (left) and VRC03-bound (right) 16055 and JRFL SOSIP. (TIF) [file ppat.1004570.s008.tif]
