# Supplementary material for: Well-Ordered Trimeric HIV-1 Subtype B and C Soluble Spike Mimetics Generated by Negative Selection Display Native-like Properties
Source: PLoS Pathog. 2015 Jan 8;11(1):e1004570. doi: 10.1371/journal.ppat.1004570 (PMC4287557; doi:10.1371/journal.ppat.1004570)
Supplement: S1 Table — Antibody neutralization of HIV-1 JRFL and 16055 and stoichiometry of selected Fabs on SOSIP trimers by EM. Antibody neutralization of HIV-1 JRFL and 16055 viral strains (Top). Fab occupancy detected by EM on JRFL and 16055 SOSIP trimers by EM (Middle). Number of particles computed for the determination of 3D EM reconstructions of JRFL and 16055 SOSIP trimers with selected Fabs. (DOCX) [file ppat.1004570.s009.docx]

| **Table S1. Neutralization and EM statistics** | | | | | | | | | |
| --- | --- | --- | --- | --- | --- | --- | --- | --- | --- |
|  | | | | | | | | | |
| **Antibody neutralization of HIV-1 JRFL and 16055 strains** | | | | | | | | | |
| Neutralization IC_50_ (µg/mL) | | | | | | | | | |
| mAb | JRFL | | 16055 | | mAb | | JRFL | | 16055 |
| VRC01* | 0.023 | | 0.023 | | PGT145* | | 0.017 | | 0.003 |
| VRC03 | 0.003 | | 0.02 | | PGT151 | | 0.013 | | 0.009 |
| PGV04 | 0.03 | | >50 | | PG16* | | 0.003 | | 0.005 |
| VRC06 | 0.061 | | >50 | | F105 | | >50 | | >50 |
| b12 | 0.014 | | >50 | | GE136 | | >50 | | >50 |
| 3BNC60 | 0.003 | | >50 | | GE148 | | >50 | | >50 |
| 2G12 | 0.05 | | >50 | | B6 | | >50 | | >50 |
| PGT121 | 0.011 | | 0.106 | | 17b | | >50 | | >50 |
| PGT128* | 0.007 | | >50 | | C11 | | >50 | | >50 |
| PGT135* | 5.99 | | >50 | | *Historical values | | | | |
|  |  | |  | | | |  | |  |
| **Binding of mAbs as observed by negative stain EM** | | | | | | | | | |
|  | | JRFL SOSIP trimer stoichiometry | | | | | | | |
| Ligand | | Trimer alone | | 1 ligand | | 2 ligands | | 3 ligands | |
| PGV04 Fab | | 8% | | 8% | | 50% | | 34% | |
| PGT151 Fab | | 25% | | 28% | | 36% | | 11% | |
| sCD4 | | 1% | | 6% | | 10% | | 83% | |
| VRC01 Fab | | 12% | | 8% | | 15% | | 65% | |
| VRC03 Fab | | 11% | | 21% | | 30% | | 38% | |
| 19b | | 75% | | 25% | | 0% | | 0% | |
|  | |  | |  | |  | |  | |
|  | | 16055 SOSIP trimer stoichiometry | | | | | | | |
| Ligand | | Trimer alone | | 1 ligand | | 2 ligands | | 3 ligands | |
| PGV04 Fab | | 38% | | 52% | | 10% | | 0% | |
| PGT151 Fab | | 46% | | 34% | | 20% | | 0% | |
| sCD4 | | 2% | | 8% | | 30% | | 52% | |
| VRC01 Fab | | 8% | | 11% | | 18% | | 63% | |
| VRC03 Fab | | 10% | | 8% | | 30% | | 52% | |
| 19b | | 78% | | 22% | | 0% | | 0% | |
|  | |  | |  | |  | |  | |
| **Number of particles used in the determination of the 3D EM models** | | | | | | | | | |
|  | | JRFL SOSIP | | | | 16055 SOSIP | | | |
| Unliganded | | 53573 | |  | | 33952 | |  | |
| sCD4 | | 20820 | |  | | 16476 | |  | |
| VRC01 | | 22762 | |  | | 8395 | |  | |
| VRC03 | | 50109 | |  | | 39514 | |  | |
| PGT151 | | 16361 | |  | | N/A | |  | |
| PGV04 | | (Day 0) 28712 | | (Day 7) 35657 | |  | |  | |
